# Development and Validation of Machine Learning Models for Postjudgment Estimation of High Compensation Ratios After Lower Limb Fracture Surgery: Retrospective Study

**DOI:** 10.2196/93801

**Published:** 2026-09-22

**Authors:** Lixuan Song, Siwen Zhao, Yuanzheng Deng, Ruqin Yang, Hongyang Wang, Shumin Zhang, Wenjun Li, Wen Wen, Zijian Lv, Hongtao Lei, Taipin Guo

**Affiliations:** 1 Yunnan University of Chinese Medicine Kunming, Yunnan China; 2 Kunming Medical University Kunming China

**Keywords:** lower limb fracture surgery, medical malpractice, compensation ratio, machine learning, post-judgment estimation

## Abstract

**Background:**

Orthopedic surgery is the second most common subspecialty involved in medical malpractice claims, wherein lower limb surgery carries a higher risk of claims and involves higher compensation amounts. However, effective tools for postjudgment estimation of high compensation ratios and consistency assessment against prior similar cases after lower limb fracture surgery are currently lacking in medicolegal risk management and judicial practice.

**Objective:**

This study aimed to develop and validate multiple machine learning (ML) models to estimate a medical malpractice compensation ratio of ≥50% in postjudgment, nonfinalized medicolegal cases, and to systematically evaluate the models’ discriminative ability, stability, calibration performance, and medicolegal utility.

**Methods:**

This study developed binary classification models based on 451 medical malpractice cases after lower limb fracture surgery in China from 2004 to 2025. Among these cases, 360 cases from eastern, northeastern, and central China constituted the development dataset and were randomly split at a 7:3 ratio into a training set (n=251) and an internal test set (n=109), whereas 91 cases from western China were held out as an independent geographical external validation set. Least Absolute Shrinkage and Selection Operator (LASSO) was used for feature selection. Logistic regression (LR), k-nearest neighbors (KNN), support vector machine (SVM), random forest (RF), and extreme gradient boosting (XGBoost) were trained using 5-fold cross-validation and grid search. Model performance was assessed using the receiver operating characteristic (ROC) curve, area under the receiver operating characteristic curve (AUROC), bootstrap resampling, calibration curves, and decision curve analysis (DCA).

**Results:**

LASSO identified following 8 predictors: inappropriate surgical procedure, age, inadequate medical records, lack of informed consent, disability severity grade 1-4, sex, treatment delay, and inadequate preoperative preparation. In the test set, the LR model achieved an AUROC of 0.933, with recall, precision, accuracy, and *F*_1_-score of 0.810, 0.940, 0.872, and 0.870, respectively. Inappropriate surgical procedure and lack of informed consent were the strongest model-associated factors. Bootstrap analysis showed stable LR discrimination, and calibration was favorable, with a Brier score of 0.105. DCA suggested a potential reference value across threshold probabilities. Considering discrimination, calibration, classification performance, parsimony, and interpretability, the LR model was selected as the final model. External validation provided preliminary support for cross-regional transportability.

**Conclusions:**

ML-based models for postjudgment estimation may provide a nonbinding historical benchmark for estimating whether a compensation ratio of ≥50% is broadly consistent with previous similar cases in medical malpractice claims. Notably, the LR model showed the most favorable overall performance among the compared models in terms of discriminative ability, stability, calibration, and medicolegal utility, providing an auxiliary postjudgment reference for hospital risk management and legal departments, legal practitioners, courts, and other judicial professionals before a case becomes final or is practically concluded.

**Trial Registration:**

OSF Registries 10.17605/OSF.IO/EMAKT; https://osf.io/emakt/overview

## Introduction

Surgery poses risks not only to patients but also to surgeons in the event of medical errors [1]. Within the medical field, surgical subspecialties are frequently the target of medical malpractice claims. Orthopedic surgery ranks as the second most common subspecialty involved in medical malpractice claims, trailing only neurosurgery, and accounts for nearly 15% of all medical malpractice cases annually [2,3]. It has been estimated that orthopedic surgeons face an average of 17 lawsuits throughout their careers [4]. Medical malpractice claims in orthopedic surgery are numerous and involve high compensation [5-7], imposing a substantial economic and time burden across the medicolegal system [8,9]. Specifically, claims related to lower limb surgery carry a higher risk [10] and involve greater compensation amounts [11,12]. However, research investigating the causes and risk factors of claims in this domain remains relatively limited. Most studies have focused on spinal surgery or elective surgeries, such as joint replacement, and have relied primarily on descriptive statistics [13-16], lacking the capacity for case-level postjudgment medicolegal reference assessment. Although machine learning (ML) has been increasingly applied in medical and health informatics [17], its applications remain scarce in the field of medical malpractice.

In recent years, the number of medical malpractice cases in China has continued to rise, making orthopedics a particularly high-incidence specialty [7,18]. However, systematic studies regarding litigation following lower limb fracture surgery remain absent in China. Therefore, the purpose of this study is to analyze these medical malpractice lawsuits. This analysis can retrospectively identify key medicolegal factors associated with orthopedic claims. Furthermore, given that legal professionals often find themselves in a predicament when facing judicial appraisal opinions due to a lack of medical and forensic knowledge [19], developing postjudgment estimation models may provide a supplementary reference for evaluating consistency with previous similar cases.

Existing studies on medical malpractice across multiple specialties have mainly described claim characteristics, allegations of negligence, compensation amounts, and litigation outcomes [1,20-22], whereas validated case-level models for postjudgment estimation and nonbinding consistency assessment of compensation ratios remain scarce. Importantly, different medical specialties and clinical environments (including outpatient care, inpatient care, diagnostic and treatment procedures, and surgery) are highly heterogeneous, placing different demands on medical professionals, and the potential medical errors also differ. Therefore, different departments and clinical environments must be considered in medical litigation research [23]. In this study, we focused on litigation cases following lower limb fracture surgery, identified and validated key medicolegal variables associated with compensation ratios of ≥50%, and developed geographically externally validated ML models for postjudgment estimation and nonbinding reference assessment before cases become final or are practically concluded. Further details on the intended postjudgment use scenario and interpretation of model outputs are provided in Multimedia Appendix 1.

## Methods

### Data Source

A search was conducted in the China Judgments Online database using the following criteria: (1) full-text search: “postoperative,” (2) document type: “judgment,” (3) cause of action: “civil cause of action—tort liability dispute—medical malpractice liability dispute,” and (4) keywords: “medical accident.” A total of 4934 cases from 2004 to 2025 were retrieved. Cases were included if they met the following criteria: (1) the patient underwent lower limb fracture surgery; and (2) the judicial appraisal opinion specified specific acts of negligence, which were subsequently adopted in the final judgment. The exclusion criteria were as follows: (1) upper limb and trunk fracture surgery, or complex orthopedic surgery involving multiple body parts; (2) joint replacement surgery; and (3) appraisal opinions that only generally stated: “the medical party was at fault.” Before applying the nonspecific opinion exclusion criterion, 509 eligible lower limb fracture surgery cases were identified; of these, 451 were included in the final analysis, and 58 were excluded.

### Ethical Considerations

This study was conducted in accordance with applicable ethical principles for research using publicly available data. It relied exclusively on legally obtained, publicly available judicial documents retrieved from the China Judgments Online database and involved no direct participant recruitment, contact, intervention, collection of biological samples, or access to nonpublic medical records. According to Article 32 of the Measures for Ethical Review of Life Science and Medical Research Involving Humans in China, research using legally obtained public data or anonymized information may be exempt from ethics review when it does not cause harm to individuals and does not involve identifiable sensitive personal information or commercial interests [24]. Therefore, no ethics committee review was sought, and no ethics approval, exemption, case, or application number was issued.

Informed consent was not obtained because no individuals were recruited, enrolled, contacted, or intervened upon, and no primary research dataset collected under informed consent was reused. The original judicial documents were published online by the courts in accordance with the Provisions of the Supreme People’s Court on the Issuance of Judgments by the People’s Courts on the Internet, which require deletion or partial replacement of specified personal information before online publication [25]. To further protect privacy and confidentiality, direct identifiers, including names, identification numbers, home addresses, contact information, medical record numbers, original case numbers, and other potentially identifying information, were not extracted into the analytic dataset or reported in the manuscript or supplementary materials. The findings are reported only in aggregate form or as deidentified model outputs. No compensation was provided because there was no participant recruitment, contact, intervention, or follow-up. All figures and supplementary materials were reviewed to ensure that they contain no identifiable individuals, facial images, original judgment screenshots, names, case numbers, medical record numbers, or other personal identifiers.

### Study Design and Study Subjects

This retrospective study aimed to develop and validate an ML model for postjudgment estimation of whether the medical compensation ratio would be ≥50% in medical malpractice cases. Data were sourced from 4 distinct geographical regions in China. Cases from the Eastern, Central, and Northeastern regions constituted the model development dataset (n=360), whereas cases from the Western region were held out a priori as an independent geographical external validation set (n=91). This geographical split was used to evaluate preliminary cross-regional transportability in an independent regional validation cohort.

After the external validation set had been separated, the 360-case development dataset was randomly partitioned at a 7:3 ratio into a training set (n=251) for model training and hyperparameter optimization and an internal test set (n=109) for internal performance evaluation. The random seed for data partitioning was fixed (random seed=1) to allow consistent data splitting during model development and evaluation. To evaluate potential observable covariate shift between datasets, baseline covariates were compared between the model development dataset and the independent geographical external validation set, with *P* values and standardized mean differences (SMDs) reported.

The study subjects were identified from medical malpractice judgments and judicial appraisal opinions in multiple regions, encompassing demographics, litigation details, and factors related to medical practice. After the rules had been prespecified and reviewed, 5 reviewers assisted with data extraction under a predefined protocol (including a lawyer and a surgeon familiar with medical malpractice). Candidate litigation-related predictors were preliminarily evaluated by the study investigators based on clinical significance and scientific knowledge. Moreover, 3 legal experts and 3 clinical experts provided professional consultation regarding medicolegal interpretation and clinical relevance of candidate variables. Finally, 23 key features were ultimately identified for subsequent analysis under the following categories: (1) demographics: sex and age; (2) litigation details: lawsuit year, lawsuit duration days, tertiary hospital, court level, accident severity grade, and disability severity grade 1-4; and (3) medicolegal findings related to clinical practice: unauthorized medical practice, improper postoperative care, inappropriate surgical procedure, inadequate medical records, lack of informed consent, suboptimal surgical outcome, treatment delay, diagnostic error, improper complication management, inadequate preoperative preparation, improper medication use, breach of clinical standards, adverse outcome related preexisting conditions, natural disease progression, and patient nonadherence. For document-dependent medicolegal variables, including inappropriate surgical procedure, inadequate preoperative preparation, inadequate medical records, and lack of informed consent, coding was assigned only when the corresponding finding was explicitly recorded in the judicial appraisal opinion and adopted in the final judgment. Region was used exclusively as the stratification criterion for external validation and was therefore excluded from the feature list.

The primary outcome variable was defined as a medical compensation ratio of ≥50% (Multimedia Appendix 1). Based on judicial appraisal findings and final judgments, a compensation ratio of ≥50% was coded as a positive event (labeled as 1), whereas a ratio of <50% was coded as a negative event (labeled as 0). This coding scheme, therefore, formulated the prediction target as a binary classification task. Continuous variables were retained, while categorical and ordinal variables were transformed into binary variables. Detailed feature descriptions and label assignments are presented in Multimedia Appendix 1 and Table S1 in Multimedia Appendix 2.

### Sample Size Considerations

Because this was a retrospective study including all eligible cases after applying the prespecified inclusion and exclusion criteria, a prospective sample size calculation was not feasible. Following Riley et al [26] and the TRIPOD reporting recommendations [27], we reported the number of outcome events in relation to the initial candidate predictors and described the procedures used to limit model complexity and evaluate potential overfitting risk. Details are provided in Multimedia Appendix 1, Table 1, and Tables S2 and S3 in Multimedia Appendix 2.

**Table 1:** Comparison of classification performance across models using the internal validation dataset.

Algorithms	AUROC^a^ (95% CI)	Accuracy	Recall	Specificity	Precision	NPV^b^	*F*_1_-score
RF^c^	0.922 (0.869-0.962)	0.835	0.759	0.922	0.917	0.770	0.831
XGBoost^d^	0.911 (0.855-0.955)	0.798	0.741	0.863	0.860	0.746	0.796
SVM^e^	0.905 (0.846-0.958)	0.780	0.690	0.882	0.870	0.714	0.770
KNN^f^	0.879 (0.817-0.934)	0.771	0.724	0.824	0.824	0.724	0.771
LR^g^	0.933 (0.883-0.972)	0.872	0.810	0.941	0.940	0.814	0.870

^a^AUROC: area under the receiver operating characteristic curve.

^b^NPV: negative predictive value.

^c^RF: random forest.

^d^XGBoost: extreme gradient boosting.

^e^SVM: support vector machine.

^f^KNN: k-nearest neighbors.

^g^LR: logistic regression.

### Sensitivity Analysis

To assess the robustness of the binary outcome definition used in the main analysis, an ordinal liability-grade sensitivity analysis was further performed. The original compensation ratio was mapped onto following 6 ordered medicolegal liability grades: no liability, minor liability, secondary liability, equal liability, major liability, and full liability. Detailed liability intervals and the rationale for classification are provided in Multimedia Appendix 1. The same preprocessing procedures and predictors from the primary binary model were retained, without additional feature selection. Ordered logistic and ordered probit regression models were then fitted and evaluated in the internal test set and external validation set using mean absolute error, root-mean-square error, adjacent-grade accuracy, and quadratic weighted κ, to examine whether the main findings were robust after preserving the ordinal liability structure.

To address potential selection bias related to the exclusion of nonspecific-opinion cases, a post hoc selection-bias sensitivity analysis was also performed. Included and excluded cases were compared using variables available in both groups, and inverse probability weighting (IPW) was used to evaluate whether observable selection differences affected the primary logistic regression (LR) model.

To assess potential temporal drift related to changes in medical practice and the legal environment, a post hoc temporal sensitivity analysis was performed using the full development cohort (n=360). Cases were stratified into legally meaningful periods, including 2004-2020 and 2021-2025, and model discrimination and calibration were compared across periods using out-of-fold predicted probabilities from the prespecified LR model. In addition, lawsuit year was reintroduced into LR models as both a post-2021 indicator and a continuous centered variable to evaluate whether temporal effects influenced the observed associations.

### Data Preprocessing

In the raw dataset, minor missing values were observed in certain variables. Specifically, the missing rates were 5% for age, 4.2% for lawsuit duration days, and 1.7% for sex, while the missing rates for the remaining variables were all less than 1%. To mitigate potential bias caused by complete-case deletion, this study used single iterative imputation based on chained equations using IterativeImputer with max_iter=10.

Specifically, Bayesian ridge regression was used for imputing continuous variables (age and lawsuit duration days), while LR was used for the categorical variable (sex). To avoid data leakage, the imputation model was fitted exclusively on the training set, and the derived parameters were directly applied to both the internal test set and the external validation set using the transform procedure, without refitting. Continuous imputed values for age and lawsuit duration days were not rounded during model development.

Given the relatively balanced distribution of positive and negative samples, data balancing techniques, such as Synthetic Minority Oversampling Technique (SMOTE), were not used. Since SMOTE was originally proposed for imbalanced classification tasks [28], synthetic oversampling was unlikely to provide substantial benefit in this study and could introduce additional noise or increase overfitting risk [29], particularly given the modest sample size.

### Feature Selection

To reduce model complexity, mitigate multicollinearity, and enhance model generalizability, Least Absolute Shrinkage and Selection Operator (LASSO) was used for feature selection. Within the training set, the optimal regularization parameter (α) was determined using 5-fold cross-validation. Both the α corresponding to the minimum cross-validation error (α_min_) and the α within 1 SE of the minimum (α_1SE_) were identified. To construct a more robust and interpretable model, the feature set corresponding to α_1SE_ was selected.

Using the α_1SE_ criterion, a total of 8 predictors were identified, including inappropriate surgical procedure, age, inadequate medical records, lack of informed consent, disability severity grade 1-4, sex, treatment delay, and inadequate preoperative preparation. These features were subsequently used to develop all ML models. Notably, both LASSO feature selection and single iterative imputation were conducted exclusively within the training set. The derived parameters were subsequently applied to the test and external validation sets to ensure the objectivity of model evaluation. In addition, variance inflation factors (VIFs) were calculated for the LASSO-selected predictors to assess potential multicollinearity among variables.

### Model Development and Evaluation

Based on the selected feature set, the following 5 ML models were developed: k-nearest neighbors (KNN), support vector machine (SVM), random forest (RF), LR, and extreme gradient boosting (XGBoost). For the primary analysis, hyperparameter optimization was performed for all models using 5-fold cross-validation combined with grid search within the training set, using the area under the receiver operating characteristic curve (AUROC) as the primary optimization metric. Model-specific hyperparameters were tuned for KNN, SVM, RF, and XGBoost, while other parameters were prespecified to control model complexity and ensure reproducibility. LR was fitted as a prespecified unpenalized LR model for interpretability and comparability. The complete search spaces, fixed parameters, and selected final settings are provided in Table S4 in Multimedia Appendix 2. The optimal parameters identified were subsequently applied to train the final models. Model performance was evaluated using multiple metrics, including AUROC, specificity, sensitivity, negative predictive value, precision (positive predictive value), Brier score, accuracy, and Matthews correlation coefficient (MCC). Bootstrap procedures for primary model performance evaluation and IPW sensitivity analyses were performed using a fixed random seed (random seed=42) to ensure reproducibility. The number of bootstrap resamples was set according to the purpose of each analysis, with 1000 resamples used for AUROC stability assessment and AUROC CI estimation and 10,000 resamples used for paired AUROC comparison. Pairwise bootstrap AUROC comparisons were performed between each complex ML model and the LR baseline. In addition, to assess whether the model comparison was affected by restrictive tuning of more flexible tree-based models, an expanded hyperparameter sensitivity analysis was performed for RF and XGBoost. This analysis used the same predefined data partition, preprocessing workflow, and analysis settings as the primary analysis (random seed=1). The original KNN and SVM settings were retained because their main model-specific hyperparameters had already been tuned. The expanded tuning settings are provided in Table S5 in Multimedia Appendix 2.

### Model Interpretability

To support interpretation of the LR model for estimating whether the medical compensation ratio was ≥50%, this study calculated adjusted odds ratios (aORs) along with their 95% CIs and *P* values. The primary aOR estimates were derived from the LR model fitted using the training cohort (n=251) after random splitting of the development dataset (n=360) into training and internal test sets. Given LASSO-based feature selection, the reported aORs, 95% CIs, and *P* values should be interpreted as post-selection exploratory estimates, not confirmatory inference. A forest plot was generated to visualize the direction and magnitude of the associations between the selected predictors and the model-estimated outcome.

Furthermore, a nomogram was constructed to provide a visual and intuitive tool for case-level medicolegal reference assessment, translating the impact of each specific predictor into a point-based system to estimate the model-based probability of a high compensation ratio for specific cases.

### Web-Based Model Deployment Using Streamlit

To improve the postjudgment medicolegal applicability of the model, we deployed the final LR model as a web application using Streamlit Python. After users enter the relevant features, the web application automatically generates a model-based reference estimate of the probability of a compensation ratio ≥50% and provides a nonbinding reference estimate rather than a binding liability determination. To improve end user usability and reduce ambiguity, the disability severity grade field is configured to accept the actual numeric grade from 1 to 10, with the prespecified binary transformation performed automatically in the backend. The tool is intended for use after a nonfinal judgment has been served but before the case becomes legally final or is practically concluded, and should not be interpreted as a real-time clinical warning system or point-of-care clinical decision-support tool, or a tool for determining the liability ratio.

The Streamlit app (Snowflake) was deployed on a managed cloud-hosted Streamlit platform as a prototype interface, without an author-maintained dedicated server or database. The web interface contains only predefined categorical options and numeric fields for deidentified model variables, with no free-text fields for names, identification numbers, medical record numbers, case numbers, addresses, or contact information; at the application level, user-entered variables are used only transiently to generate the model estimate and are not stored, retained by the authors, or used for model retraining.

### Statistical Analysis

To describe the baseline characteristics of the study population and compare the differences between compensation ratio groups, baseline analysis was performed on continuous and categorical variables, respectively. Continuous variables included age, lawsuit duration days, and lawsuit year. The Shapiro-Wilk test was used to assess the normality of the variable distribution. Normally distributed variables were expressed as mean (SD), and intergroup comparisons were conducted using the independent samples *t* test. Nonnormally distributed variables were presented as median (IQR), and the Mann-Whitney *U* test was used for intergroup comparisons. Categorical variables were expressed as frequencies and percentages. For 2×2 contingency tables, Fisher exact test was used when any expected cell count was less than 5, whereas the chi-square test was used for all other cases. Data analysis was performed using Python (version 3.8.0; Python Software Foundation). All statistical tests were 2-tailed, and statistical significance was set at *P*<.05.

## Results

### Study Population

Following the application of inclusion and exclusion criteria, 451 eligible cases were included in the final analysis. Cases from three regions constituted the development dataset (n=360), which was randomly split at a 7:3 ratio into a training set (n=251) and an internal test set (n=109). Cases from the fourth region were reserved as an independent geographical external validation set (n=91). The study selection process and dataset allocation are shown in Figure 1.

**Figure 1:**
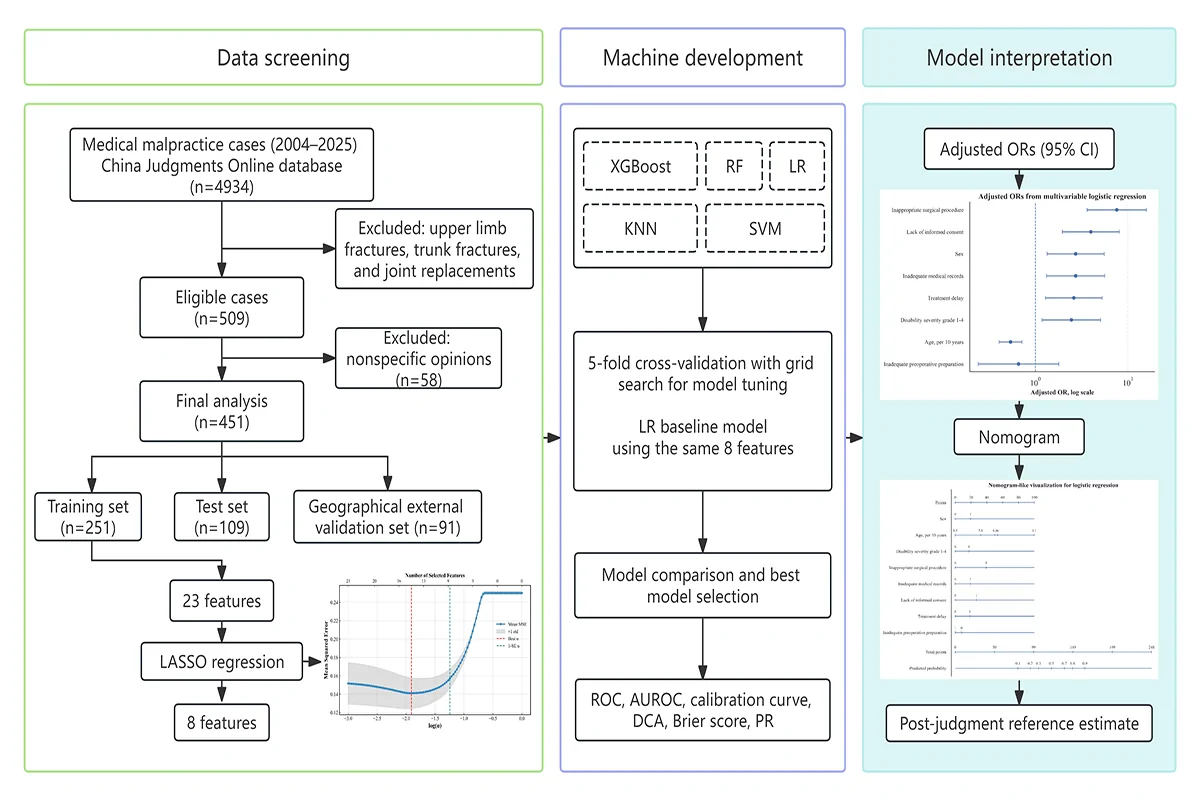
Study flowchart. Following the application of inclusion and exclusion criteria, 451 eligible cases were included. Cases from 3 regions were assigned to the development dataset (n=360), which was then randomly split at a 7:3 ratio into a training set (n=251) and an internal test set (n=109), whereas cases from the fourth region were reserved as an independent external validation set (n=91). Least Absolute Shrinkage and Selection Operator regression was used for feature selection. 5 machine learning models were developed and evaluated, and the best-performing model was selected for interpretability analysis. AUROC: area under the receiver operating characteristic curve; DCA: decision curve analysis; KNN: k-nearest neighbors; LASSO: Least Absolute Shrinkage and Selection Operator; LR: logistic regression; OR: odds ratio; PR: precision-recall; ROC: receiver operating characteristic; RF: random forest; SVM: support vector machine; XGBoost: extreme gradient boosting.

### Baseline Characteristics

A total of 360 medical malpractice cases were included in the development dataset, comprising 186 (51.7%) cases with a compensation ratio of ≥50% and 174 (48.3%) cases with a ratio of <50%. Significant differences were observed between the 2 groups regarding multiple demographic characteristics, litigation details, and medicolegal findings related to clinical practice (Table S6 in Multimedia Appendix 2).

In terms of continuous variables, cases in the compensation ratio ≥50% group involved younger patients than those in the <50% group (median 40.0, IQR 38.5-58.0 y vs median 58.0, IQR 40.0-58.0 y; *Z*=7.17; *P*<.001), and lawsuit duration days were also significantly shorter (median 174.0, IQR 100.5-422.0 d vs median 233.0, IQR 133.0-426.8 d; *Z*=2.05; *P*=.04). No significant difference was observed in the distribution of lawsuit year between the groups (*P*=.83).

In terms of categorical variables, significant differences were observed between the 2 groups regarding sex, disability severity grade 1-4, tertiary hospital, inappropriate surgical procedure, inadequate medical records, lack of informed consent, and treatment delay (all *P*<.05). Notably, inappropriate surgical procedure, inadequate medical records, and lack of informed consent were significantly more prevalent in the compensation ratio ≥50% group.

### Feature Selection

LASSO was performed for feature selection within the training set. Cross-validation results indicated that at the minimum cross-validation error (α_min_=.012), the model retained a larger number of features. Conversely, applying the 1-SE criterion (α_1SE_=.057) yielded a sparser and more stable model (Figure S1 in Multimedia Appendix 3). The dynamic shrinkage of coefficients during this process is illustrated in Figure S2 in Multimedia Appendix 3.

Based on the α_1SE_ criterion, 8 features were ultimately selected (Figure S3 in Multimedia Appendix 3), including inappropriate surgical procedure (positive coefficient), age (negative coefficient), inadequate medical records (positive coefficient), lack of informed consent (positive coefficient), disability severity grade 1-4 (positive coefficient), sex (positive coefficient), treatment delay (positive coefficient), and inadequate preoperative preparation (slight negative coefficient). Although inadequate preoperative preparation was retained as a selected feature by the LASSO procedure, its slightly negative coefficient indicates that it should not be interpreted as a positively associated predictor in the LASSO model. Among these selected features, inappropriate surgical procedure exhibited the largest positive coefficient, whereas age showed a negative coefficient. Overall, the LASSO procedure identified a parsimonious set of variables with potential medicolegal relevance for subsequent model development. The VIFs for all LASSO-selected predictors ranged from 1.02 to 1.11, indicating no evidence of severe multicollinearity (Table S7 in Multimedia Appendix 2).

### Model Performance Comparison

Model evaluation encompassed following 4 dimensions: discrimination, precision-recall (PR) performance, calibration, and medicolegal utility. Relevant results are summarized in Figure 2. Within the test set, all 5 models exhibited robust discrimination. Notably, the LR model achieved the highest AUROC (0.933), with recall, precision, accuracy, and *F*_1_-score of 0.810, 0.940, 0.872, and 0.870, respectively. Overall, the LR model demonstrated the most favorable overall performance for predicting a compensation ratio of ≥50%.

**Figure 2:**
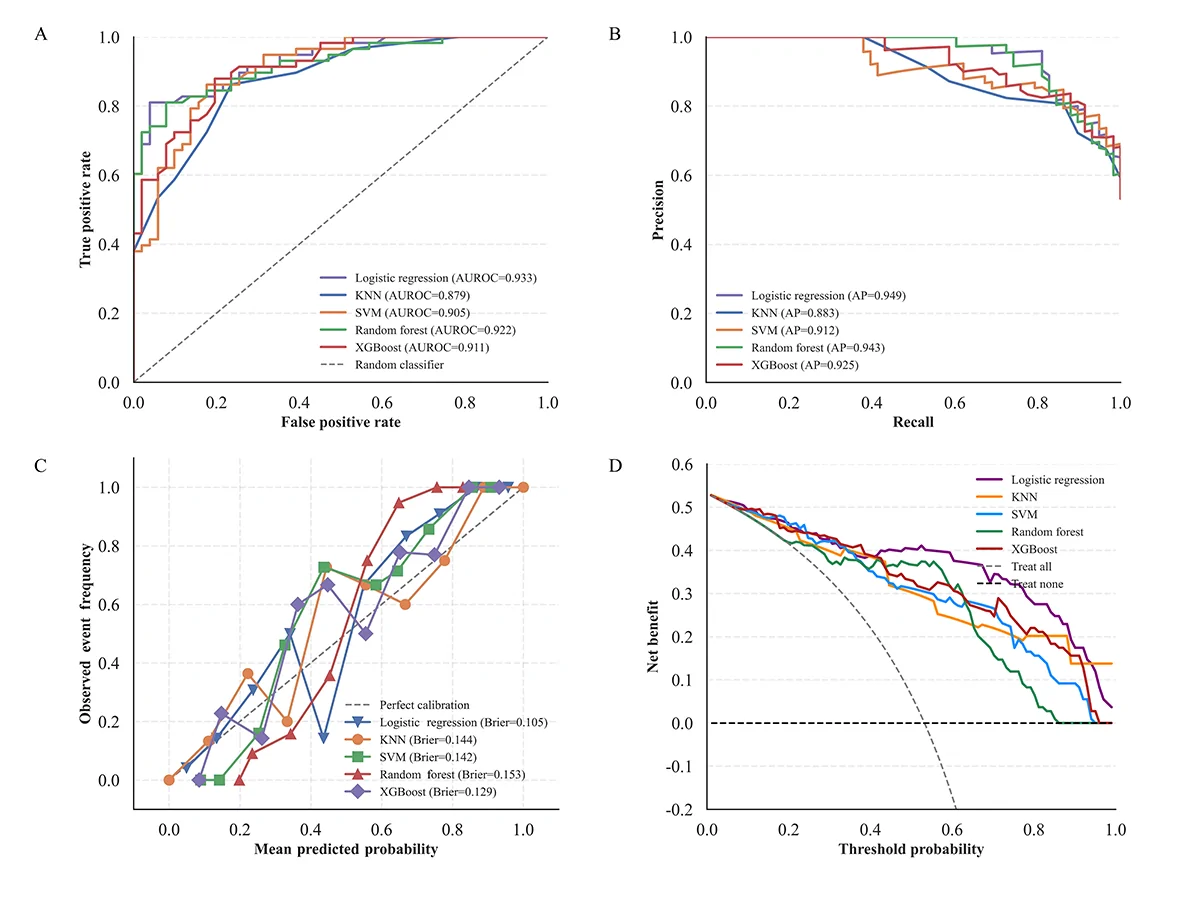
Model performance on the internal test set. (A) receiver operating characteristic (ROC) curves of the 5 ML models, (B) precision-recall (PR) curves, (C) calibration curves, and (D) decision curve analysis (DCA). AUROC: area under the receiver operating characteristic curve; KNN: k-nearest neighbors; SVM: support vector machine; XGBoost: extreme gradient boosting.

Given the study’s focus on estimating the likelihood of a high compensation ratio in postjudgment cases, PR curves were used for further model evaluation. Results indicated that the LR model achieved the highest average precision (0.949), with relatively high precision across most recall thresholds. This suggests favorable performance for identifying cases with a compensation ratio of ≥50%.

The calibration curve of the LR model most closely approximated the perfect calibration line, accompanied by the lowest Brier score (0.105), which indicates optimal agreement between predicted probabilities and observed outcomes. Furthermore, decision curve analysis (DCA) demonstrated that the LR model generally achieved higher net benefit across a wide range of threshold probabilities, suggesting favorable potential for postjudgment estimation and reference use.

In addition to graphical analysis, multiple standard classification performance metrics were computed, including accuracy, sensitivity (recall), specificity, positive predictive value (precision), negative predictive value, *F*_1_ score, and AUROC (Table 1).

None of the complex ML models significantly outperformed the LR baseline in pairwise bootstrap AUROC comparisons, as illustrated in Table S8 in Multimedia Appendix 2.

Taken together with the receiver operating characteristic (ROC) curve, PR, calibration, DCA, and quantitative metric analyses, the **LR model demonstrated the most favorable overall performance** and was therefore selected as the final model for subsequent external validation and interpretability analysis.

This conclusion was further examined in an expanded hyperparameter sensitivity analysis for RF and XGBoost, in which broader tuning improved their AUROC values compared with the original tuning settings but did not yield a consistent overall advantage over LR. Detailed results are provided in Tables S9-S10 in Multimedia Appendix 2 and Figure S4 in Multimedia Appendix 3.

### Model Stability Analysis

To evaluate the stability of model discriminative performance, 1000 bootstrap resamples were performed on the test set with a fixed random seed (random seed=42) to calculate the AUROC distributions and 95% CIs for each model (Figure 3). The bootstrapping results indicated that the LR model achieved a mean AUROC of 0.932 (95% CI 0.883-0.972), surpassing the other 4 models. Furthermore, its AUROC distribution was relatively concentrated, suggesting good model stability. Conversely, the KNN model exhibited a relatively dispersed AUROC distribution, reflecting lower stability. These findings further suggested that the LR model not only performed optimally in the single test set but also maintained robust discriminative ability under repeated sampling conditions.

**Figure 3:**
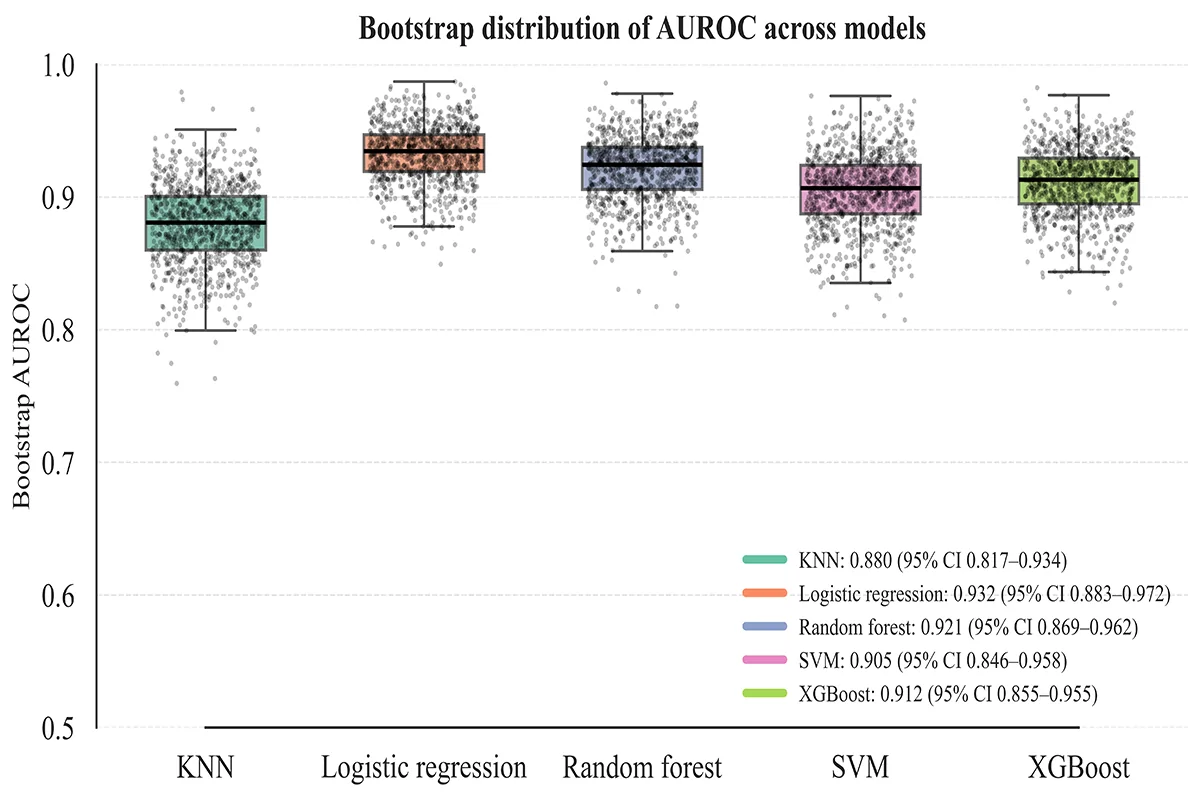
Evaluation of model discriminative stability. The figure displays the distribution of AUROC values and 95% CIs derived from 1000 bootstrap resamples on the internal test set for the KNN, SVM, random forest, XGBoost, and logistic regression models. AUROC: area under the receiver operating characteristic curve; KNN: k-nearest neighbors; SVM: support vector machine; XGBoost: extreme gradient boosting.

### External Validation

To further evaluate the model’s generalizability and robustness across different regions, this study used a geographical external validation strategy. Specifically, medical dispute data from 4 distinct regions were used. Data from 3 regions were allocated for model training and parameter optimization, while the remaining region served as a completely independent external validation set to evaluate model performance under a cross-regional validation setting. The external validation set comprised 91 cases, with baseline characteristics detailed in Table S11 in Multimedia Appendix 2. A baseline comparison between the model development dataset and the independent geographical external validation set is presented in Table S12 in Multimedia Appendix 2. The measured baseline covariates showed no major imbalance between the development and external validation datasets, with all SMDs below 0.20. However, this finding only indicates no severe imbalance in measured variables and does not exclude unmeasured regional heterogeneity in health care delivery, judicial appraisal, compensation practices, litigation behavior, or court reporting practices. External validation results indicated that the LR model exhibited favorable performance across the evaluation metrics, showing acceptable performance (Table S3 in Multimedia Appendix 2 and Figure S5 in Multimedia Appendix 3).

In addition, MCC was calculated as a supplementary classification metric. As presented in Table S13 in Multimedia Appendix 2, the LR model achieved the highest MCC in both the internal test set and the geographical external validation set, indicating consistent classification performance across the 2 evaluation datasets.

### Predictor Analysis and Model Interpretability

The LR model provided explicit and quantifiable estimates for each selected feature. As visually summarized in the forest plot (Figure 4), inappropriate surgical procedure showed the strongest positive association with the model outcome (aOR 7.60, 95% CI 3.63-15.89; *P*<.001). Similarly, lack of informed consent (aOR 3.98, 95% CI 1.96-8.08; *P*<.001), sex (aOR 2.73, 95% CI 1.34-5.56; *P*=.006), inadequate medical records (aOR 2.73, 95% CI 1.32-5.64; *P*=.007), treatment delay (aOR 2.61, 95% CI 1.29-5.29; *P*=.008), and disability severity grade 1-4 (aOR 2.45, 95% CI 1.18-5.08; *P*=.02) were also positively associated with the predicted outcome.

**Figure 4:**
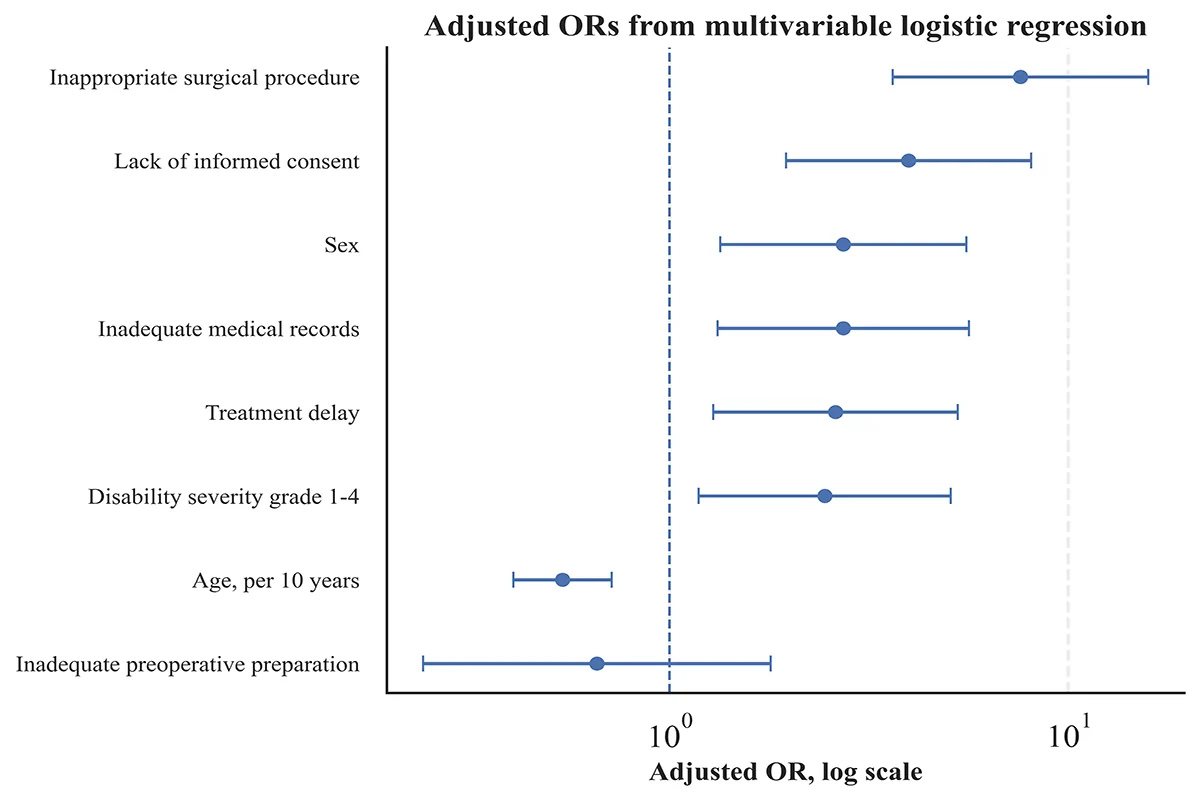
Logistic regression forest plot. Dots indicate adjusted odds ratios, and horizontal bars indicate 95% CIs for predictors of a compensation ratio ≥50%. The adjusted ORs were derived from the primary logistic regression model fitted using the training cohort (n=251) after randomly splitting the development dataset (n=360) into training and internal test sets. OR: odds ratio.

Conversely, older age showed an inverse association (aOR 0.54 per 10-year increase, 95% CI 0.41-0.72; *P*<.001), indicating that older patients had lower model-estimated odds of a high compensation ratio. Notably, inadequate preoperative preparation did not show a statistically significant independent association (aOR 0.66, 95% CI 0.24-1.79; *P*=.41). Consistent with its slight negative LASSO coefficient, this variable was not interpreted as a predictor associated with increased odds of the outcome.

To further support case-level postjudgment estimation, a nomogram-like visualization was constructed based on the LR model (Figure 5). This visualization translates each predictor into a point-based score and links the total score to the predicted probability of a compensation ratio ≥50%, thereby providing an intuitive representation of the model-based medicolegal reference estimate.

**Figure 5:**
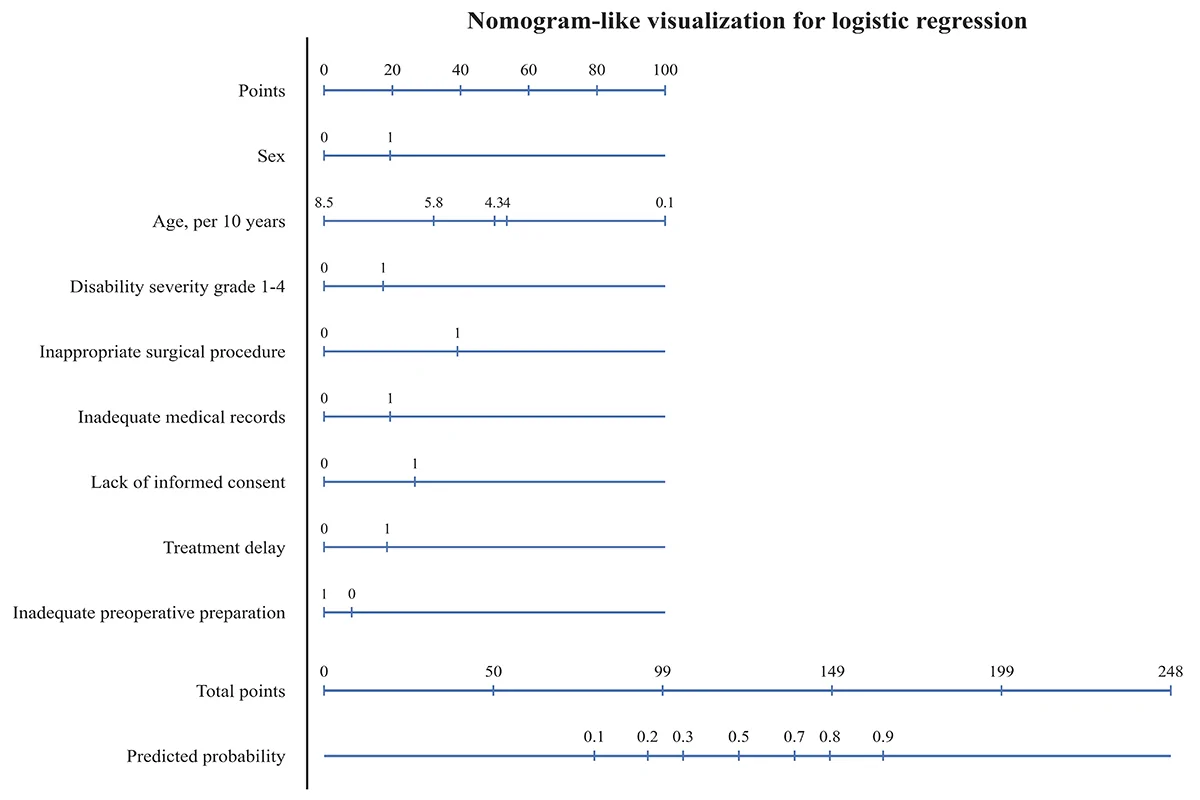
Logistic regression nomogram. Each predictor is assigned a point score, and the total points correspond to the predicted probability of a compensation ratio ≥50%.

### Sensitivity Analysis

In the ordinal liability-grade sensitivity analysis, ordered logistic and ordered probit models showed predictor directions consistent with the main binary model. The ordered logistic model achieved adjacent-grade accuracies of 0.789 and 0.780 in the internal validation set and external validation set, respectively, with quadratic weighted κ values of 0.455 and 0.477. Similar results were observed for the ordered probit model. Detailed results are provided in Tables S14-S16 in Multimedia Appendix 2. These findings support the robustness of the main conclusions while indicating limited exact 6-grade classification accuracy, thereby supporting the use of the legally meaningful ≥50% binary end point as the primary modeling target.

In the selection-bias sensitivity analysis, included and excluded cases were generally comparable across most observed variables; however, regional distribution differed significantly between groups, and the largest preweighting imbalances were observed for Eastern China, Central China, and the court level. The normalized stabilized IPW had a narrow distribution, with a mean of 1.000 (SD 0.060; range 0.928-1.173) and an effective sample size of 449.4. Model performance remained nearly unchanged after IPW adjustment, with internal test AUROCs of 0.933, 0.934, and 0.934 and external validation AUROCs of 0.915, 0.915, and 0.917 for the primary unweighted, IPW-weighted, and IPW-refit analyses, respectively. IPW analyses suggested limited sensitivity of AUROC estimates to measured selection differences, although residual covariate imbalance and potential unmeasured selection bias should be considered when interpreting these findings. Detailed results are provided in Tables S17-S19 in Multimedia Appendix 2 and Figure S6 in Multimedia Appendix 3.

In the temporal sensitivity analysis, model performance was comparable between the 2004-2020 and 2021-2025 periods, with AUROCs of 0.903 and 0.902 and Brier scores of 0.131 and 0.125, respectively. Reintroducing temporal variables into the LR model showed that neither the post-2021 indicator nor the continuous lawsuit year was significantly associated with a high compensation ratio. These findings suggest that the primary model results were not materially affected by temporal drift or major legal transitions during the study period. Detailed results are provided in Tables S20 and S21 in Multimedia Appendix 2 and Figures S7 and S8 in Multimedia Appendix 3.

### Development of a Web-Based Postjudgment Estimation Tool

To present the finalized model in an accessible format, an interactive Streamlit-based web application was developed. The tool allows users to input case-level medicolegal variables after a nonfinal judgment has been served and obtain a model-based reference estimate of the probability of a compensation ratio ≥50%. The interface is illustrated in Figure S9 in Multimedia Appendix 3. This application should be interpreted as a prototype for postjudgment estimation and nonbinding reference use. Formal usability testing, assessment of integration into postjudgment administrative and legal review workflows, cybersecurity audit, and evaluation of the tool’s potential impact on review-related decision-making were not performed in this study.

## Discussion

### Principal Findings

To the best of our knowledge, this is the first study to apply ML-based classification modeling to characterize case-level predictors associated with high compensation ratios after lower limb fracture surgery. In this study, we analyzed and compared cases with a compensation ratio of <50% with those with a ratio of ≥50%. Subsequently, 8 key features were selected using LASSO, and predictive models were constructed using 5 distinct ML algorithms, with their performance evaluated via multiple metrics. The LR model exhibited optimal performance and was selected as the final model. Furthermore, a web-based prototype was developed to present case-level postjudgment reference estimates. The intended users include hospital risk management departments, hospital legal departments, legal practitioners, courts, and other judicial professionals.

Inadequate medical records, inappropriate surgical procedure, lack of informed consent, sex, age, disability severity grade 1-4, and treatment delay were identified as model-based predictors associated with a compensation ratio ≥50% following lower limb fracture surgery. These findings are consistent with previous research and should be interpreted as predictive associations rather than causal effects. In contrast, although inadequate preoperative preparation was retained during LASSO feature selection, it showed a slight negative coefficient and was not statistically significant in the final LR interpretability analysis. Therefore, it was not interpreted as a key risk-increasing predictor.

Successful defense in medical malpractice litigation depends on evidentiary documentation rather than factual assertions alone. Incomplete, missing, ambiguous, or invalid documentation can render it impossible to refute the plaintiff’s allegations, and consequently, some defensible cases are lost due to insufficient evidence [30]. The implementation of electronic health records may influence the litigation process by increasing the amount of documentation available to defend against or substantiate medical malpractice claims [31]. Given that many lawsuits hinge on submitting accurate documentation to the court, comprehensive and accurate medical records are essential for medical professionals to effectively navigate litigation [32].

Previous studies of surgical-related claims have reported inappropriate surgical procedures as a leading allegation in malpractice lawsuits [33-37]. A systematic review and meta-analysis reported an association between surgical technical skills and clinical outcomes [38].

A retrospective study indicated that claims arising from the failure of medical professionals to properly fulfill their obligation to provide patients with comprehensive information accounted for 14% of all judgments in the field of medical malpractice liability [39]. Previous research has suggested that providing patients with adequate and clear preoperative information may be associated with a lower probability of litigation [40]. Placing greater emphasis on communication and patient comprehension may represent the simplest and most effective strategy to improve outcomes and mitigate litigation risks [41]. Beyond enhancing awareness of the legal implications of informed consent, previous literature has emphasized the importance of optimizing patient communication regarding surgical indications, diagnosis, treatment, and outcomes for reducing unnecessary litigation, enhancing patient outcomes, and mitigating the burden of defensive medicine [42].

In a study investigating medical malpractice lawsuits following traumatic fractures, 59.7% of the plaintiffs were male [43]. Furthermore, a Westlaw-based demographic analysis of orthopedic malpractice claims indicated that male sex served as a significant negative predictor of verdicts favorable to the defendant [44].

Medical malpractice cases are associated with insufficient professional competence and repeated failures (lack of therapeutic effect), and functional and sensory losses are assessed by peers. Medical law limits personal injury caused by illegal procedures, and medical regulations define the boundaries of medical procedures related to personal injury caused by illegal operations, provided that a causal relationship between such injury and the behavior of medical professionals is established. Some studies indicate that postoperative functional impairment is a potential area for medical malpractice lawsuits and may involve high compensation amounts [45-47].

Research on cases involving inferior vena cava filters indicated that the most common complaints were treatment refusal or delay (accounting for 35% of cases) [48]. Similarly, a study of ophthalmology telemedicine-related lawsuits within the Westlaw database noted that 94.5% of the cases alleged delays in assessment and/or treatment [49].

The inverse direction observed for inadequate preoperative preparation should not be interpreted as a protective effect. The outcome of this study was a compensation ratio of ≥50%, reflecting medicolegal attribution rather than adverse clinical outcomes per se. Inadequate preoperative preparation is an upstream and broad negligence category. After more proximate factors, such as inappropriate surgical procedure, were included in the model, its independent association with high compensation may have been attenuated. This interpretation is consistent with methodological evidence showing that adjustment for intermediate or downstream proxy variables can alter the apparent association between an upstream factor and an outcome [50]. This pattern is also compatible with methodological discussions showing that the magnitude or direction of an association may change after adjustment for another variable in a multivariable model [51]. Therefore, the slight negative and statistically nonsignificant coefficient should be regarded as a model-adjusted estimate after accounting for other selected predictors, rather than evidence of an independent risk-reducing or protective effect. In addition, the post hoc VIF analysis did not indicate severe multicollinearity among the selected predictors, suggesting that the inverse direction was unlikely to be explained by strong linear collinearity [52]. Nevertheless, given that the coefficient was small and statistically nonsignificant, no substantive protective interpretation should be assigned to this model-adjusted estimate.

Older age was inversely associated with a compensation ratio ≥50%. This may be related to the physical condition of older adult patients. Previous studies have indicated that, in general, older adult patients file fewer claims [53]. A study on the risk of osteoporosis and fracture after solid organ transplantation revealed that among different age groups, patients aged more than 61 years exhibited the highest risk of osteoporosis and fracture [54]. Relevant legal literature also provides support for this. According to the argument by Lewis et al [55] on the multiplier effect, younger patients have a longer expected working life and more years of remaining life, which corresponds to a significantly higher compensation base. Meanwhile, Geistfeld [56] noted that juries should consider the “duration of injury” when assessing injury severity. Younger patients, due to their longer life expectancy, are deemed in legal logic to endure a greater total amount of pain over their remaining lifespan. Therefore, the trend identified by the model may reflect the emphasis in judicial practice on compensation for loss of labor capacity, as well as the potential tendency to more fully recognize the consequences of damages sustained by young victims.

By defining the outcome as a compensation ratio of ≥50%, this study identified model-based predictors associated with a high compensation ratio in lower limb fracture litigation, and is primarily intended to support postjudgment estimation and nonbinding consistency assessment in cases where a nonfinal judgment has been served on the parties but has not yet taken legal effect, or the dispute has not otherwise been finally resolved. Medical malpractice litigation is often time-consuming and costly because determining fault, liability, and compensation involves complex and uncertain legal assessments [57]. In this context, the model provides an auxiliary way to place the current compensation ratio in the context of historical patterns from prior similar cases.

Beyond specific predictors, the model may serve as a supplementary, nonbinding historical reference tool during postjudgment case evaluations. By translating case information into an estimated probability of a high compensation ratio, the tool may help hospital risk management departments, hospital legal departments, legal practitioners, courts, and other judicial professionals consider whether the current compensation ratio is broadly consistent with historical patterns in prior similar cases. When a substantial discrepancy is observed, the model output should be interpreted as a signal for contextual review rather than as evidence that the current liability allocation is incorrect. Practical application must remain case-specific, and absolute reliance on the algorithm should be avoided.

For hospital administration, the model may function as a practical postjudgment risk management and financial preparation tool. After a nonfinal judgment has been served on the parties, and before the judgment has taken legal effect or the dispute has otherwise been finally resolved, hospital risk management and legal departments may use the model output to support compensation reserve assessment, insurance communication, litigation-resource planning, and institution-level risk improvement. However, the estimate should be interpreted together with case-specific legal analysis, judicial appraisal findings, and the full evidentiary record.

For legal and judicial practice, the model may provide a consistency-checking reference by comparing the compensation ratio reflected in a nonfinal judgment or decision with historical patterns in similar cases. Substantial discrepancies may prompt legal practitioners or courts to further examine the reasoning underlying appraisal-opinion adoption, liability-ratio allocation, compensation-item calculation, and individualized case circumstances. Nevertheless, the model output should remain supplementary and should not replace judicial appraisal, legal reasoning, judicial discretion, or evidentiary assessment.

However, the deployment of ML within the judicial system necessitates prudent consideration of multiple ethical issues [58,59]. First, algorithmic bias may arise when prediction models are trained on data that reflect uneven case selection, institutional practice patterns, or jurisdiction-specific decision environments. In this study, this concern is particularly relevant because judicial records may vary by case publication practices, appraisal standards, regional litigation patterns, and compensation practices. Therefore, subgroup performance, calibration, and error patterns should be evaluated before broader use [60,61]. Although this study has enhanced transparency through interpretability analysis, interpretability alone does not establish accountability. For institutional use, responsibility for model validation, updating, deployment, and human interpretation should be clearly defined, and model outputs should remain auditable rather than be treated as self-justifying conclusions [62]. In the context of this study, the model should therefore be used only as an auxiliary reference rather than as a substitute for judicial appraisal, legal reasoning, or case-specific evidentiary assessment [63,64]. Second, automation bias refers to the tendency of human users to overrely on AI outputs. In the context of this web-based probability tool, this risk may arise if users give excessive weight to numerical estimates without sufficient consideration of judicial appraisal, legal reasoning, and case-specific evidence [65]. For real-world implementation, robust regulatory frameworks and accountability mechanisms must be established to ensure the transparency and legal compliance of algorithmic decision-making and to safeguard against overreliance on algorithms that could compromise judicial fairness [60,62-65].

### Limitations

First, considering the heterogeneity of medical specialties and clinical scenarios, the sample size was relatively small, potentially introducing selection bias, although this was a prerequisite to ensure the accuracy of the results. Second, due to variations in medical practices and legal systems across countries, transnational external validation was not performed, limiting the model’s generalizability. Third, this study relied on publicly available judicial documents, and database coverage may be affected by court publication practices and the completeness of case documentation. Cases that were not publicly available or lacked sufficient information for variable extraction and outcome determination could not be systematically included, which may limit the generalizability of the model to broader medicolegal disputes.

This limitation is particularly relevant to the interpretation of model-derived probabilities in real-world postjudgment reference use. Because the model was developed only from cases that ultimately generated publicly available judicial decisions, it did not capture disputes resolved before or outside public adjudication, such as through mediation, settlement, or withdrawal, or disputes that remained unresolved. This selection mechanism may have led the dataset to overrepresent cases with greater injury severity, stronger legal contestation, more complete documentation, or a higher likelihood of proceeding to formal adjudication. Consequently, the model-derived probabilities may not be directly generalizable to the broader population of lower limb fracture malpractice disputes, particularly less severe or less contested cases that are resolved without a public judgment. Even within the intended postjudgment use scenario, when the model is applied to postjudgment cases that have not yet become final and that involve less severe injury, less contested liability issues, or less complete evidentiary records, uncritical reliance on the model-estimated probability may overestimate the likelihood of a high compensation ratio or reduce the reliability and calibration of the reference estimate. Therefore, model outputs should be interpreted as historical reference estimates learned from publicly adjudicated cases rather than as calibrated probabilities applicable to all real-world medicolegal disputes.

In addition, formal interrater reliability statistics were not available for the extracted medicolegal variables. Although missingness was assumed to be approximately missing at random because the missing rates were low, nonrandom missingness related to systematic legal reporting differences cannot be completely excluded.

### Future Work

Future work should proceed in 2 directions. First, implementation-oriented studies are needed to evaluate the usability, integration into postjudgment administrative and legal review workflows, cybersecurity, and potential impact on review-related decision-making of the current postjudgment reference tool among hospital risk management departments, hospital legal departments, legal practitioners, courts, and other judicial professionals. Such studies should examine whether model outputs can improve review consistency, workflow efficiency, or decision quality without increasing automation bias, and should further assess subgroup performance, fairness, calibration drift, privacy protection, and cybersecurity before institutional deployment. Second, future model-development studies should conduct temporal validation using newly adjudicated cases collected within China’s medicolegal framework under consistent inclusion criteria and variable definitions. Future studies should also incorporate independent duplicate data extraction and report interrater reliability for key medicolegal variables. Given variations in medical practices and legal systems across countries, future research should develop and validate jurisdiction-specific models tailored to different medicolegal contexts. In addition, because this study focused only on malpractice cases after lower limb fracture surgery, future research may develop separate specialty-specific medicolegal models for postjudgment estimation and reference assessment for disputes in other medical specialties.

### Conclusions

This study used KNN, SVM, RF, LR, and XGBoost for modeling. Among the compared models, LR was selected as the best-performing model among the evaluated algorithms. Although the model established in this study is currently only applicable to postjudgment estimation and nonbinding reference assessment of high compensation ratios after lower limb fracture surgery, we have demonstrated the feasibility of this method. This may provide a useful framework for hospital risk management, legal review, compensation reserve assessment, insurance communication, postjudgment case review, and nonbinding consistency assessment before cases become final or are practically concluded.

## Appendix

Multimedia Appendix 1 Definitions and methodological details, including the intended post-judgment use and interpretation of model outputs, variable definitions, primary outcome definition, sample size considerations, and the definition of the ordinal liability-grade outcome.

Multimedia Appendix 2 Supplementary tables, including feature coding and label assignment, model performance across datasets, hyperparameter and expanded tuning settings, baseline characteristics of the development and external validation sets, variance inflation factor assessment for selected predictors, bootstrap AUROC and MCC analyses, ordinal liability-grade sensitivity analyses, inverse probability weighting analyses, and temporal subgroup analyses.

Multimedia Appendix 3 Supplementary figures, including LASSO feature selection and coefficient plots, ROC curves from expanded hyperparameter sensitivity analyses, external validation performance plots, inclusion probability and inverse probability weighting distributions, temporal subgroup ROC and calibration curves, and the interface of the web-based post-judgment medicolegal reference assessment tool.

## Abbreviations

aOR: adjusted odds ratio
AUROC: area under the receiver operating characteristic curve
DCA: decision curve analysis
IPW: inverse probability weighting
KNN: k-nearest neighbors
LASSO: Least Absolute Shrinkage and Selection Operator
LR: logistic regression
MCC: Matthews correlation coefficient
ML: machine learning
PR: precision-recall
RF: random forest
ROC: receiver operating characteristic
SMD: standardized mean difference
SMOTE: Synthetic Minority Oversampling Technique
SVM: support vector machine
VIF: variance inflation factor
XGBoost: extreme gradient boosting

## Data Availability

The datasets used or analyzed during this study are not publicly available to minimize potential reidentification risks and confidentiality concerns related to case-level medicolegal information. The full source code is not publicly available because it is linked to the restricted case-level dataset and the web-based implementation workflow. Relevant analytical code or analysis scripts may be made available from the corresponding author upon reasonable request, subject to approval by the corresponding author or research team and any applicable data-sharing requirements.
