# Supplementary material for: Development and Validation of Machine Learning Models for Postjudgment Estimation of High Compensation Ratios After Lower Limb Fracture Surgery: Retrospective Study
Source: JMIR Med Inform. 2026 Sep 22;14:e93801. doi: 10.2196/93801 (PMC13643259; doi:10.2196/93801)
Supplement: Supplementary file 1 [file medinform_v14i1e93801_app1.pdf]

Supplementary Materials

Post-judgment Use and Output Interpretation..2

Variable definitions...3

Outcome variable definition...4

Sample Size Considerations...7

Definition of the ordinal model outcome..8

Post-judgment Use and Output Interpretation

This model is not intended for real-time clinical prediction, clinical decision-making, or determining the legal liability ratio. Instead, it is designed as a non-binding historical benchmark comparison tool for post-judgment medicolegal risk assessment.

The intended application scenario is limited to post-judgment cases in which a non-final judgment or decision containing a compensation-ratio determination has already been formally served on the parties, but the case has not yet become final or been practically concluded. In this study, a non-final judgment or decision served on the parties refers to a procedural stage in which the parties have received a court judgment or decision reflecting a compensation-ratio determination, while the case remains within the statutory period for appeal, objection, reconsideration, or other available procedural review, or has not otherwise reached a final procedural endpoint under the applicable rules.

The phrase “the dispute has not otherwise been finally resolved” refers to situations in which the case has not reached a practical or procedural endpoint through other mechanisms, such as appellate adjudication, court-mediated settlement, out-of-court settlement, withdrawal of appeal, withdrawal of claims, or full performance of a judgment, mediation agreement, or settlement agreement.

Within this limited post-judgment and pre-finalization context, the model output should be interpreted as an estimated probability that the compensation ratio is ≥50% based on historical patterns in prior similar publicly adjudicated cases. The model is intended to help users place the compensation ratio reflected in a current non-final judgment or decision within the broader context of historical case patterns. It does not replace judicial appraisal, legal reasoning, judicial discretion, or case-specific evidentiary assessment.

The intended users include hospital risk management departments, hospital legal departments, legal practitioners, courts, and other judicial professionals. For hospital-related users, the model may provide a supplementary reference for

compensation reserve assessment, insurance communication, and institutional risk management. For legal and judicial users, it may provide a non-binding reference for comparing the compensation ratio reflected in a current non-final judgment or decision with historical patterns in prior similar cases.

Both concordant and discordant model outputs may have practical reference value. When the model estimate is broadly consistent with the compensation ratio reflected in the current non-final judgment or decision, it may support the view that the current ratio is generally aligned with historical patterns in similar publicly adjudicated cases. When an obvious discrepancy is observed, the model output should not be interpreted as evidence that the current judgment, decision, or liability allocation is incorrect. Rather, it may serve as a signal for contextual review of factors such as the adoption of judicial appraisal opinions, liability-ratio allocation, compensation-item calculation, individualized case circumstances, and the evidentiary record.

The model output should remain non-binding and should not be used as an independent basis for dispute resolution, settlement, mediation, appeal strategy, or judicial decision-making.

Variable definitions

This study included medical malpractice liability dispute cases from 30 provincial-level administrative divisions in China. According to China's regional economic classification [1], these areas are divided into four major geographical regions: Eastern, Central, Western, and Northeastern China. As a pioneer of reform and opening-up, Eastern China leads in economic development. Central China ranks as the nation's second most developed economic region, and Western China exhibits a relatively lower level of economic development. Northeastern China comprises only three provinces: Liaoning, Jilin, and Heilongjiang.

Hospital tier classification standards are established in accordance with China's Hospital Tier Classification Management Specifications [2]. These specifications assess hospital accreditation tiers by evaluating facility scale, staffing, medical

hardware, research capabilities, and technical proficiency. Hospitals are categorized into three tiers: (1) Class I hospitals: community-level institutions providing preventive care, medical treatment, health services, and rehabilitation directly to local communities; (2) Class II hospitals: district/county-level hospitals offering comprehensive medical services to multiple communities and undertaking specific teaching and research functions; (3) Class III hospitals: supra-regional hospitals providing high-level specialized medical services across multiple regions and undertaking higher education and research responsibilities. All Class I, II, and III hospitals are publicly funded and administered, with their comprehensive capabilities progressively enhancing with ascending tier levels.

According to the Classification Standards for Medical Malpractice (Trial Implementation) [3], medical impairment grades in China are classified into ten levels. The categories encompass: "Mild Impairment" (Grades 10-7, characterized by partial loss of self-care capacity), "Moderate Impairment" (Grades 6-5, involving significant loss of self-care capacity), "Severe Impairment" (Grades 4-1, resulting in complete loss of self-care capacity), "Death," and "Serious Medical Condition" (denoting deterioration in the patient's condition without leading to permanent impairment).

According to the Classification Standards for Medical Malpractice (Trial Implementation) [4], medical malpractice incidents in China are classified into four grades, each defined by the degree of patient harm sustained: Grade 1 involves patient death or severe permanent impairment; Grade 2 encompasses moderate permanent impairment or organ/tissue damage causing severe functional impairment; Grade 3 comprises mild permanent impairment or organ/tissue damage resulting in moderate functional impairment; and Grade 4 pertains to incidents causing evident personal injury, yet without resulting in permanent impairment or severe functional impairment.

Outcome variable definition

The compensation ratio in this study was not treated as a purely monetary variable,

but as an operational representation of court-adopted medicolegal responsibility after judicial appraisal and adjudication. In China, the Guideline for Judicial Appraisal of Medical Damage (SF/T 0097-2021), issued by the Ministry of Justice, provides the medical-damage-specific appraisal framework for evaluating medical fault, damage consequences, causation, and the degree of responsibility. Under this framework, liability levels or causative force are classified into six ordered categories: full responsibility, major responsibility, equal responsibility, secondary responsibility, slight responsibility, and no responsibility [5].

Given the uncertainty of causation in medical malpractice cases, Chinese courts widely apply the principle of proportional liability to allocate responsibility [6]. Judicial appraisal opinions may describe fault participation in three forms: specifying a liability level, such as major responsibility; providing a specific numerical value; or indicating a percentage range, such as 25%-35% [7]. Regardless of the descriptive form used in the appraisal opinion, the final compensation amount is calculated by multiplying the total compensable loss by a determinate percentage, namely the compensation ratio. Therefore, the compensation ratio reflects the court-adopted proportional allocation of medicolegal responsibility rather than the absolute compensation amount alone.

The 50% threshold was selected as an operational and legally interpretable cutoff rather than as a clinical cutoff or a statutory rule. Although SF/T 0097-2021 provides the medical damage appraisal framework and the six-level responsibility structure, it does not prescribe a fixed numerical compensation percentage for each responsibility level. This is consistent with the principle that the final allocation of compensation remains within judicial discretion. To provide a more concrete numerical basis for the 50% cutoff used in this study, we further referred to empirical evidence from Chinese medical damage liability judgments. Dong's empirical study summarized the commonly used judicial responsibility intervals as follows: 100% for full responsibility, 60%-90% for major responsibility, 40%-60% for equal responsibility, 20%-40% for secondary responsibility, and 0%-20% for slight responsibility. Notably,

among cases in which the appraisal opinion identified equal responsibility, courts adopted 50% as the compensation responsibility proportion in 130 of 147 cases, accounting for 88.4% [8].

Accordingly, a compensation ratio of ≥50% was defined as the primary high-compensation-ratio endpoint because it identifies cases in which the court-adopted liability proportion reached at least the conventional midpoint of equal responsibility or entered a higher responsibility range. A lower cutoff, such as 40%, was not used because 40% lies at the boundary between secondary responsibility and the lower range of equal responsibility. Using 40% as the positive endpoint would include borderline cases in which the medical provider's responsibility had only reached the lower margin of equal responsibility. Conversely, a higher cutoff, such as 60%, would correspond to the boundary between equal responsibility and major responsibility and would exclude cases within the upper range of equal responsibility. By contrast, 50% corresponds to the conventional midpoint of equal responsibility and has been frequently adopted by courts when equal responsibility is identified. Therefore, the ≥50% threshold was used as a more conservative and interpretable operational endpoint for identifying cases with relatively high court-adopted medicolegal responsibility.

This endpoint is therefore not an arbitrary statistical dichotomization, but a pragmatic medicolegal outcome based on the Chinese medical damage appraisal framework and empirical adjudication practice. A simple plaintiff win/loss endpoint was not used because Chinese medical malpractice judgments commonly involve proportional compensation rather than an all-or-none allocation of liability. Coding any compensation as a plaintiff win would have produced a highly imbalanced outcome, as cases with no compensation awarded to the plaintiff were relatively uncommon in our dataset. Therefore, the compensation ratio was used to construct a legally interpretable binary endpoint for model development. It should be emphasized that the ≥50% cutoff was adopted solely as an operational outcome definition for model development and should not be interpreted as implying a legally dichotomous

structure of medical damage liability. In individual cases, the final compensation ratio remains subject to the court's comprehensive assessment of the appraisal opinion, the factual circumstances of the case, and other relevant evidence.

To further address the concern that dichotomization may oversimplify the graded structure of medicolegal liability, we additionally constructed an ordinal liability-grade outcome as a sensitivity analysis. This ordinal endpoint was designed to preserve the ordered nature of court-adopted responsibility allocation and to examine whether the modeling results remained robust when the compensation outcome was modeled as a graded medicolegal construct rather than as a binary high-compensation-ratio endpoint.

Sample Size Considerations

Because this was a retrospective study based on all eligible judicial cases after applying the prespecified inclusion and exclusion criteria, a prospective sample size calculation before data collection was not feasible.

According to Riley et al. (2019) [9] and the reporting recommendations of the TRIPOD guidelines [10], sample size adequacy was assessed by comparing the number of outcome events with the number of candidate predictors. In the development dataset, 360 cases were available, including 186 positive events and 174 negative events. A total of 23 candidate predictors were initially considered. Under the commonly cited rule of at least 10 events per candidate predictor, 230 positive events would have been required if all predictors were entered directly into an unpenalized model. In this dataset, the events per candidate predictor would have been 8.1 based on positive events and 7.6 based on the smaller outcome category, indicating a potential risk of overfitting.

To reduce this risk, LASSO feature selection was performed exclusively within the training set using the 1-standard-error criterion, reducing the final feature set from 23 to 8 predictors. In addition, 5-fold cross-validation combined with grid search was conducted within the training set (n=251) for hyperparameter optimization, using

AUROC as the primary optimization metric. The final LR model showed relatively stable discriminative performance across the training, internal test, and external validation sets, with AUROC values of 0.900, 0.933, and 0.915, respectively. The maximum absolute AUROC difference was 0.033, which was less than 4 percentage points. Bootstrap resampling of the internal test set also yielded a mean AUROC of 0.932 (95% CI 0.883-0.972), providing additional evidence of relatively stable discriminative performance. Nevertheless, because the overall sample size and the external validation cohort remained modest, potential overfitting could not be completely excluded, and further validation in larger datasets is warranted.

Definition of the ordinal model outcome

For the ordinal liability-grade sensitivity analysis, the original compensation ratio was mapped onto six ordered medicolegal responsibility grades to preserve the graded structure of liability allocation. This outcome setting was based on the responsibility-degree framework reflected in the Guideline for Judicial Appraisal of Medical Damage (SF/T 0097-2021) [5] and the empirical responsibility intervals summarized by Dong [8] in Chinese medical damage liability judgments. Specifically, 0% was coded as no responsibility; >0% to ≤20% as slight responsibility; >20% to ≤40% as secondary responsibility; >40% to ≤60% as equal responsibility; >60% to ≤90% as major responsibility; and >90% to ≤100% as full responsibility. The 0% category was retained separately because it represents the absence of court-adopted compensation responsibility for the medical provider, which is conceptually distinct from slight but still positive responsibility. To avoid overlap between adjacent categories at boundary values, compensation ratios greater than 0% were classified using a left-open and right-closed interval structure. Under this coding rule, 100% was naturally included in the full-responsibility category.

References

1. Wang Q. The Evolution of Regional Economic Disparities in China. China Foreign Investment Review. 2023(11):50-3.

2. Du C. An Empirical Analysis of China's Hospital Grading Management System. Social Sciences Frontline. 2018(04):84-94.

3. Zhang C, Du Z. An Analysis of Disability Assessment Standards in Medical Malpractice Liability Disputes. Chinese Forensic Science. 2014(01):106-9.

4. Medical Malpractice Classification Standards (Trial Implementation). New Regulations Monthly. 2003(09):33-8.

5. Ministry of Justice of the People's Republic of China. Guideline for Judicial Appraisal of Medical Damage: SF/T 0097-2021 Beijing: Ministry of Justice of the People's Republic of China; 2021.

6. Yu X. Causal Uncertainty in Chinese Medical Malpractice Law - When Theories Meet Facts. Tsinghua China Law Review. 2016;Vol. 9, No. 1.

7. Wei Y, Cheng W. An empirical study on the current situation of judicial appraisal in medical malpractice liability disputes in Sichuan Province: an analysis based on samples from China Judgments Online in 2019. Chinese Journal of Forensic Sciences. 2020(6):80-5. doi: 10.3969/j.issn.1671-2072.2020.06.013.

8. Dong C. Challenges on the Viewpoint on Replacement of Litigation by Forensic Appraisal in Liability of Medical Damages Chinese Journal of Forensic Sciences. 2024(1):91-8. doi: 10.3969/j.issn.1671-2072.2024.013.

9. Riley RD, Snell KI, Ensor J, Burke DL, Harrell FE, Jr., Moons KG, et al. Minimum sample size for developing a multivariable prediction model: PART II - binary and time-to-event outcomes. Stat Med. Mar 30 2019;38(7):1276-96. PMID: 30357870. doi: 10.1002/sim.7992.

10. Collins GS, Reitsma JB, Altman DG, Moons KG. Transparent Reporting of a multivariable prediction model for Individual Prognosis or Diagnosis (TRIPOD): the TRIPOD statement. Ann Intern Med. Jan 6 2015;162(1):55-63. PMID: 25560714. doi: 10.7326/m14-0697.
