# Supplementary material for: Development and Validation of Machine Learning Models for Postjudgment Estimation of High Compensation Ratios After Lower Limb Fracture Surgery: Retrospective Study
Source: JMIR Med Inform. 2026 Sep 22;14:e93801. doi: 10.2196/93801 (PMC13643259; doi:10.2196/93801)
Supplement: Supplementary file 2 [file medinform_v14i1e93801_app2.pdf]

Supplementary Tables

Table S1 Features and label assignment...2
Table S2 Comparison of the prediction results of each model using the training dataset...3
Table S3 Comparison of the prediction results of each model using the external validation dataset...4
Table S4 Hyperparameter search and final settings...5
Table S5 Expanded hyperparameter settings...6
Table S6 Characteristics of the two groups of cases...8
Table S7 Variance inflation factors for LASSO-selected predictors...12
Table S8 Bootstrap AUROC Comparison vs LR...13
Table S9 Internal test set performance after expanded hyperparameter tuning...14
Table S10 External validation set performance after expanded hyperparameter tuning...15
Table S11 Characteristics of the two groups of cases (External Validation Set)...16
Table S12 Baseline Characteristics of the Development and External Validation Sets...20
Table S13 MCC results across datasets...24
Table S14 Grade Distribution...25
Table S15 Performance Metrics of Ordinal Liability-Grade Sensitivity Models...26
Table S16 Predictor Effects...27
Table S17 Included vs Excluded Cases...28
Table S18 IPW Diagnostics...29
Table S19 IPW Sensitivity Results...30
Table S20 Temporal Subgroup Performance...31
Table S21 Logistic regression models with temporal adjustment...32

Supplementary Table S1. Features and label assignment.

Name	Assignments
Accident severity grade[a]	Grade 1/2=1; Grade 3/4=0
Court level[b]	Supreme/High People's Court=1; Else=0
Region[c]	Eastern China=1; Northeastern China=2; Central China=3; Western China=4
Sex	Male=1; Female=0
Disability severity grade[d]	Grade 1-4=1; Grade 5-10=0
Tertiary hospital[e]	Yes=1; No=0
Inappropriate surgical procedure	Yes=1; No=0
Improper postoperative care	Yes=1; No=0
Inadequate medical records	Yes=1; No=0
Lack of informed consent	Yes=1; No=0
Suboptimal surgical outcome	Yes=1; No=0
Treatment delay	Yes=1; No=0
Diagnostic error	Yes=1; No=0
Improper complication management	Yes=1; No=0
Inadequate preoperative preparation	Yes=1; No=0
Unauthorized medical practice	Yes=1; No=0
Improper medication use	Yes=1; No=0
Breach of clinical standards	Yes=1; No=0
Adverse outcome-related preexisting conditions	Yes=1; No=0
Natural disease progression	Yes=1; No=0
Patient non-adherence	Yes=1; No=0

[a]Accident severity grade: Grade 1-2 (severe/moderate permanent impairment or death)=1; Grade 3-4 (mild/no permanent impairment)=0.

[b]Court level: Supreme People's Court & High People's Courts=1; Intermediate & Primary People's Courts=0.

[c]Region: Eastern China=1; Northeastern China=2; Central China=3; Western China=4.

[d]Disability severity grade: Grade 1-4 (severe impairment, complete loss of self-care ability, death/serious condition)=1; Grade 5-10 (moderate or mild impairment, significant or partial loss of self-care ability)=0.

[e]Tertiary hospital: Class III hospital=1; Class I or II hospital=0.

Other binary features (Inappropriate surgical procedure, Improper postoperative care, etc.): Presence of the specific medical error or factor=1; Absence=0.

Supplementary Table S2. Comparison of the prediction results of each model using the training dataset.

Algorithms	AUROC[a]	Accuracy	Recall	Specificity	Precision	NPV[b]	F1 Score
RF[c]	0.882	0.809	0.766	0.854	0.845	0.778	0.803
XGBoost[d]	0.940	0.853	0.867	0.837	0.847	0.858	0.857
SVM[e]	0.956	0.908	0.914	0.902	0.907	0.910	0.911
KNN[f]	0.893	0.797	0.797	0.797	0.803	0.790	0.800
LR[g]	0.900	0.789	0.789	0.789	0.795	0.782	0.792

[a]AUROC: area under the receiver operating characteristic curve.

[b]NPV: negative predictive value.

[c]RF: random forest.

[d]XGBoost: extreme gradient boosting.

[e]SVM: support vector machine.

[f]KNN: k-nearest neighbors.

[g]LR: logistic regression.

Supplementary Table S3. Comparison of the prediction results of each model using the external validation dataset.

Algorithms	AUROC[a]	Accuracy	Recall	Specificity	Precision	NPV[b]	F1 Score
RF[c]	0.905	0.813	0.714	0.929	0.921	0.736	0.805
XGBoost[d]	0.888	0.769	0.694	0.857	0.850	0.706	0.764
SVM[e]	0.882	0.747	0.633	0.881	0.861	0.673	0.729
KNN[f]	0.841	0.725	0.673	0.786	0.786	0.673	0.725
LR[g]	0.915	0.857	0.776	0.952	0.950	0.784	0.854

[a]AUROC: area under the receiver operating characteristic curve.

[b]NPV: negative predictive value.

[c]RF: random forest.

[d]XGBoost: extreme gradient boosting.

[e]SVM: support vector machine.

[f]KNN: k-nearest neighbors.

[g]LR: logistic regression.

Supplementary Table S4. Hyperparameter search and final settings.

Algorithms	Hyperparameter	Search space / values tested	Selected value	Type
LR[a]	penalty	None / unpenalized	None / unpenalized	Fixed
LR	solver	lbfgs	lbfgs	Fixed
LR	max_iter	5000	5000	Fixed
KNN[b]	n_neighbors	3, 5, 7, 9	9	Tuned
KNN	weights	uniform, distance	uniform	Tuned
KNN	p	1, 2	1	Tuned
SVM[c]	C	1, 10, 20	10	Tuned
SVM	gamma	0.001, 0.01, 0.1, 1	0.1	Tuned
SVM	kernel	rbf	rbf	Fixed
SVM	probability	True	True	Fixed
RF[d]	max_features	1, 2, 3, 4, 5, 6, 7, 8, 9	1	Tuned
RF	n_estimators	20	20	Fixed
RF	max_depth	3	3	Fixed
XGBoost[e]	n_estimators	10, 20, 30, 40, 50, 60, 70, 80, 90	10	Tuned
XGBoost	max_depth	3, 4, 5, 6, 7	4	Tuned
XGBoost	objective	binary: logistic	binary: logistic	Fixed
XGBoost	nthread	10	10	Fixed

[a]LR: logistic regression.
[b]KNN: k-nearest neighbors.
[c]SVM: support vector machine.
[d]RF: random forest.
[e]XGBoost: extreme gradient boosting.
Hyperparameters marked as “Tuned” were optimized using five-fold cross-validation with grid search in the training set, with the area under the receiver operating characteristic curve as the primary optimization metric. Parameters marked as “Fixed” were prespecified before model fitting and were not included in the grid search. A fixed random seed was used to ensure reproducibility.

Supplementary Table S5. Expanded hyperparameter settings.

Algorithms	Hyperparameter	Search space/values tested	Selected value	Type
LR[a]	penalty	None / unpenalized	None / unpenalized	Fixed
LR	solver	lbfgs	lbfgs	Fixed
LR	max_iter	5000	5000	Fixed
KNN[b]	n_neighbors	3, 5, 7, 9	9	Tuned
KNN	weights	uniform, distance	uniform	Tuned
KNN	p	1, 2	1	Tuned
SVM[c]	C	1, 10, 20	10	Tuned
SVM	gamma	0.001, 0.01, 0.1, 1	0.1	Tuned
SVM	kernel	rbf	rbf	Fixed
SVM	probability	True	True	Fixed
RF[d]	max_features	sqrt, log2, None, 1, 2, 3, 4	log2	Tuned
RF	n_estimators	100, 200, 500	100	Tuned
RF	max_depth	2, 3, 4, 5, None	5	Tuned
RF	min_samples_split	2, 5, 10	2	Tuned
RF	min_samples_leaf	1, 2, 5, 10	1	Tuned
RF	bootstrap	True	True	Fixed
XGBoost[e]	n_estimators	20, 50, 100, 200, 300	300	Tuned
XGBoost	max_depth	1, 2, 3, 4, 5	1	Tuned
XGBoost	objective	binary: logistic	binary: logistic	Fixed
XGBoost	nthread	10	10	Fixed
XGBoost	learning_rate	0.01, 0.03, 0.05, 0.1, 0.2	0.2	Tuned
XGBoost	subsample	0.7, 0.8, 0.9, 1.0	0.8	Tuned
XGBoost	colsample_bytree	0.7, 0.8, 0.9, 1.0	0.7	Tuned
XGBoost	min_child_weight	1, 3, 5, 10	1	Tuned
XGBoost	gamma	0, 0.1, 0.5, 1	0.5	Tuned
XGBoost	reg_alpha	0, 0.01, 0.1, 1	0.01	Tuned
XGBoost	reg_lambda	1, 3, 5, 10	5	Tuned

[a]LR: logistic regression.
[b]KNN: k-nearest neighbors.
[c]SVM: support vector machine.
[d]RF: random forest.
[e]XGBoost: extreme gradient boosting.

Hyperparameters marked as “Tuned” were optimized using five-fold cross-validation within the training set. RF was optimized using grid search, whereas XGBoost was optimized using randomized search with 150 iterations because of its larger

hyperparameter space. The area under the receiver operating characteristic curve was used as the primary optimization metric. Parameters marked as “Fixed” were prespecified and not included in the search. The original KNN and SVM settings were retained because their main model-specific hyperparameters had already been tuned. The expanded sensitivity analysis was targeted to RF and XGBoost, whose original settings were more restrictive and involved fixed or omitted complexity-related hyperparameters.

Supplementary Table S6. Characteristics of the two groups of cases.

Variable	All patients	Compensation ratio <50%	Compensation ratio ≥50%	Statistic	*P value*
N(%)	360 (100)	174 (48.3)	186 (51.7)		
Age(years)	49.5 (40.0, 58.0)	58.0 (40.0, 58.0)	40.0 (38.5, 58.0)	7.17	<.001
Lawsuit duration days	222.3 (120.0, 423.0)	233.0 (133.0, 426.8)	174.0 (100.5, 422.0)	2.05	.04
Lawsuit year	2016.0 (2009.0, 2023.0)	2011.5 (2009.0, 2023.0)	2019.0 (2009.0, 2023.0)	-0.19	.83
Accident severity grade (%)				0.86	.35
Grade 1/2	53 (14.7)	22 (12.6)	31 (16.7)		
Grade 3/4	307 (85.3)	152 (87.4)	155 (83.3)		
Court level (%)				1.18	.28
yes	146 (40.6)	65 (37.4)	81 (43.5)		
no	214 (59.4)	109 (62.6)	105 (56.5)		
Sex (%)				21.30	<.001
male	189 (52.5)	69 (39.7)	120 (64.5)		
female	171 (47.5)	105 (60.3)	66 (35.5)		
Disability severity grade (%)				31.25	<.001
Grade 1-4	180 (50.0)	60 (34.5)	120 (64.5)		
Grade 5-10	180 (50.0)	114 (65.5)	66 (35.5)		
Tertiary hospital (%)				12.35	<.001
yes	138 (38.3)	50 (28.7)	88 (47.3)		

no	222 (61.7)	124 (71.3)	98 (52.7)		
Inappropriate surgical procedure (%)				69.05	<.001
yes	190 (52.8)	52 (29.9)	138 (74.2)		
no	170 (47.2)	122 (70.1)	48 (25.8)		
Improper postoperative care (%)				6.39	.01
yes	181 (50.3)	75 (43.1)	106 (57.0)		
no	179 (49.7)	99 (56.9)	80 (43.0)		
Inadequate medical records (%)				39.28	<.001
yes	170 (47.2)	52 (29.9)	118 (63.4)		
no	190 (52.8)	122 (70.1)	68 (36.6)		
Lack of informed consent (%)				38.34	<.001
yes	194 (53.9)	64 (36.8)	130 (69.9)		
no	166 (46.1)	110 (63.2)	56 (30.1)		
Suboptimal surgical outcome (%)				1.99	.16
yes	299 (83.1)	139 (79)	160 (86.0)		
no	61 (16.9)	35 (20.1)	26 (14.0)		
Treatment delay (%)				17.62	<.001
yes	187 (51.9)	70 (40.2)	117 (62.9)		
no	173 (48.1)	104 (59.8)	69 (37.1)		
Diagnostic error (%)				1.78	.18
yes	48 (13.3)	28 (16.1)	20 (10.8)		

no	312 (86.7)	146 (83.9)	166 (89.2)		
Improper complication management (%)				3.79	.051
yes	146 (40.6)	61 (35.1)	85 (45.7)		
no	214 (59.4)	113 (64.9)	101 (54.3)		
Inadequate preoperative preparation (%)				2.11	.15
yes	53 (14.7)	31 (17.8)	22 (11.8)		
no	307 (85.3)	143 (82.2)	164 (88.2)		
Unauthorized medical practice (%)				0.08	.78
yes	59 (16.4)	30 (17.2)	29 (15.6)		
no	301 (83.6)	144 (82.8)	157 (84.4)		
Improper medication use (%)				0.00	>.99
yes	49 (13.6)	24 (13.8)	25 (13.4)		
no	311 (86.4)	150 (86.2)	161 (86.6)		
Breach of clinical standards (%)				.00	>.99
yes	62 (17.2)	30 (17.2)	32 (17.2)		
no	298 (82.8)	144 (82.8)	154 (82.8)		
Adverse outcome-related preexisting conditions (%)				0.76	.38
yes	47 (13.1)	26 (14.9)	21 (11.3)		
no	313 (86.9)	148 (85.1)	165 (88.7)		
Natural disease progression (%)				2.01	.16

yes	38 (10.6)	23 (13.2)	15 (8.1)		
no	322 (89.4)	151 (86.8)	171 (91.9)		
Patient non-adherence (%)				1.86	.17
yes	44 (12.2)	26 (14.9)	18 (9.7)		
no	316 (87.8)	148 (85.1)	168 (90.3)		

Values in the "Statistic" column represent χ^2 values for categorical variables and *Z* values (Mann-Whitney *U* test) for continuous variables.

Supplementary Table S7. Variance inflation factors for LASSO-selected predictors.

Predictor	VIF[a]	Tolerance
Age	1.111	0.900
Inadequate medical records	1.099	0.910
Disability severity grade 1-4	1.093	0.915
Lack of informed consent	1.081	0.925
Inappropriate surgical procedure	1.063	0.941
Treatment delay	1.039	0.962
Sex	1.035	0.966
Inadequate preoperative preparation	1.020	0.980

[a]VIF: variance inflation factor. Tolerance was calculated as 1/VIF. All VIF values were close to 1, indicating no evidence of severe multicollinearity among the LASSO-selected predictors.

Supplementary Table S8. Bootstrap AUROC Comparison vs LR.

Comparison	Δ AUROC[a] vs LR[b]	95% CI[c]	*P value*
KNN[d] vs LR	-0.054	-0.095 to -0.017	.005
SVM[e] vs LR	-0.028	-0.080 to 0.019	.25
RF[f] vs LR	-0.011	-0.043 to 0.017	.46
XGBoost[g] vs LR	-0.021	-0.054 to 0.007	.14

[a]ΔAUROC was calculated as AUROC of the comparator model minus AUROC of logistic regression. Negative values indicate that logistic regression achieved a higher AUROC. Pairwise comparisons were performed using bootstrap resampling of paired AUROC differences.
[b]LR: logistic regression.
[c]CI: confidence interval.
[d]KNN: k-nearest neighbors.
[e]SVM: support vector machine.
[f]RF: random forest.
[g]XGBoost: extreme gradient boosting.

Supplementary Table S9. Internal test set performance after expanded hyperparameter tuning.

Algorithms	AUROC[a] (95%CI[b])	Accuracy	Recall	Specificity	Precision	NPV[c]	F1 Score	MCC[d]	Brier score	AP[e]
RF[f]	0.925 (0.872-0.965)	0.798	0.741	0.863	0.860	0.746	0.796	0.605	0.122	0.941
XGBoost[g]	0.935 (0.886-0.973)	0.844	0.828	0.863	0.873	0.815	0.850	0.689	0.106	0.950
SVM[h]	0.905 (0.846-0.958)	0.780	0.690	0.882	0.870	0.714	0.769	0.578	0.146	0.912
KNN[i]	0.879 (0.817-0.934)	0.771	0.724	0.824	0.824	0.724	0.771	0.548	0.144	0.883
LR[j]	0.933 (0.883-0.972)	0.872	0.810	0.941	0.940	0.814	0.870	0.753	0.105	0.949

[a]AUROC: area under the receiver operating characteristic curve.

[b]CI: confidence interval.

[c]NPV: negative predictive value.
[d]MCC: Matthews correlation coefficient.
[e]AP: average precision.
[f]RF: random forest.
[g]XGBoost: extreme gradient boosting.
[h]SVM: support vector machine.
[i]KNN: k-nearest neighbors.
[j]LR: logistic regression.

Supplementary Table S10. External validation set performance after expanded hyperparameter tuning.

Algorithms	AUROC[a] (95%CI[b])	Accuracy	Recall	Specificity	Precision	NPV[c]	F1 Score	MCC[d]	Brier score	AP[e]
RF[f]	0.909 (0.842-0.960)	0.769	0.694	0.857	0.850	0.706	0.764	0.553	0.134	0.931
XGBoost[g]	0.916 (0.855-0.964)	0.824	0.796	0.857	0.867	0.783	0.830	0.651	0.119	0.937
SVM[h]	0.882 (0.810-0.944)	0.747	0.633	0.881	0.861	0.673	0.729	0.524	0.157	0.894
KNN[i]	0.841 (0.762-0.913)	0.725	0.673	0.786	0.786	0.673	0.725	0.459	0.166	0.850
LR[j]	0.915 (0.853-0.962)	0.857	0.776	0.952	0.950	0.784	0.854	0.731	0.119	0.937

[a]AUROC: area under the receiver operating characteristic curve.

[b]CI: confidence interval.

[c]NPV: negative predictive value.

[d]MCC: Matthews correlation coefficient.

[e]AP: average precision.

[f]RF: random forest.

[g]XGBoost: extreme gradient boosting.

[h]SVM: support vector machine.

[i]KNN: k-nearest neighbors.

[j]LR: logistic regression.

Supplementary Table S11. Characteristics of the two groups of cases (External Validation Set).

Variable	All patients	Compensation ratio <50%	Compensation ratio ≥50%	Statistic	*P value*
N(%)	91 (100)	42 (46.2)	49 (53.8)		
Age(years)	52.4 (40.0, 58.0)	58.0 (40.0, 58.0)	40.0 (36.0, 58.0)	3.58	<.001
Lawsuit duration days	174.0 (100.0, 378.0)	231.0 (121.0, 378.0)	136.0 (82.0, 378.0)	1.37	.17
Lawsuit year	2009.0 (2009-2023)	2009.0 (2009-2023)	2019.0 (2009-2023)	-1.10	.22
Accident severity grade (%)				-	.37
Grade 1/2	13 (14.3)	4 (9.5)	9 (18.4)		
Grade 3/4	78 (85.7)	38 (90.5)	40 (81.6)		
Court level (%)				0.36	.55
yes	41 (45.1)	17 (40.5)	24 (49.0)		
no	50 (54.9)	25 (59.5)	25 (51.0)		
Sex (%)				4.09	.04
male	44 (48.4)	15 (35.7)	29 (59.2)		
female	47 (51.6)	27 (64.3)	20 (40.8)		
Disability severity grade (%)				8.70	.003
Grade 1-4	40 (44.0)	11 (26.2)	29 (59.2)		
Grade 5-10	51 (56.0)	31 (73.8)	20 (40.8)		
Tertiary hospital (%)				4.84	.03
yes	36 (39.6)	11 (26.2)	25 (51.0)		
no	55 (60.4)	31 (73.8)	24 (49.0)		

Inappropriate surgical procedure (%)				20.14	<.001
yes	48 (52.7)	11 (26.2)	37 (75.5)		
no	43 (47.3)	31 (73.8)	12 (24.5)		
Improper postoperative care (%)				1.19	.28
yes	50 (54.9)	20 (47.6)	30 (61.2)		
no	41 (45.1)	22 (52.4)	19 (38.8)		
Inadequate medical records (%)				9.35	.002
yes	45 (49.5)	13 (31.0)	32 (65.3)		
no	46 (50.5)	29 (69.0)	17 (34.7)		
Lack of informed consent (%)				5.56	.02
yes	50 (54.9)	17 (40.5)	33 (67.3)		
no	41 (45.1)	25 (59.5)	16 (32.7)		
Suboptimal surgical outcome (%)				0.80	.37
yes	76 (83.5)	33 (78.6)	43 (87.8)		
no	15 (16.5)	9 (21.4)	6 (12.2)		
Treatment delay (%)				0.85	.36
yes	47 (51.6)	19 (45.2)	28 (57.1)		
no	44 (48.4)	23 (54.8)	21 (42.9)		
Diagnostic error (%)				0.00	>.99
yes	12 (13.2)	6 (14.3)	6 (12.2)		
no	79 (86.8)	36 (85.7)	43 (87.8)		

Improper complication management (%)				0.12	.73
yes	44 (48.4)	19 (45.2)	25 (51.0)		
no	47 (51.6)	23 (54.8)	24 (49.0)		
Inadequate preoperative preparation (%)				2.96	.09
yes	16 (17.6)	11 (26.2)	5 (10.2)		
no	75 (82.4)	31 (73.8)	44 (89.8)		
Unauthorized medical practice (%)				-	.21
yes	12 (13.2)	8 (19.0)	4 (8.2)		
no	79 (86.8)	34 (81.0)	45 (91.8)		
Improper medication use (%)				0.80	.37
yes	17 (18.7)	10 (23.8)	7 (14.3)		
no	74 (81.3)	32 (76.2)	42 (85.7)		
Breach of clinical standards (%)				1.34	.25
yes	18 (19.8)	11 (26.2)	7 (14.3)		
no	73 (80.2)	31 (73.8)	42 (85.7)		
Adverse outcome-related preexisting conditions (%)				-	.06
yes	12 (13.2)	9 (21.4)	3 (6.1)		
no	79 (86.8)	33 (78.6)	46 (93.9)		
Natural disease progression (%)				-	.04
yes	10 (11.0)	8 (19.0)	2 (4.1)		

no	81 (89.0)	34 (81.0)	47 (95.9)		
Patient non-adherence (%)				-	.13
yes	13 (14.3)	9 (21.4)	4 (8.2)		
no	78 (85.7)	33 (78.6)	45 (91.8)		

Values in the "Statistic" column represent χ^2 values for categorical variables and *Z* values (Mann-Whitney *U* test) for continuous variables.

Supplementary Table S12. Baseline Characteristics of the Development and External Validation Sets.

Variable	Model development dataset (n=360)	Independent external validation set (n=91)	*P value*	SMD
N	360	91		
Age, median (IQR)	49.5 (40.0, 58.0)	52.4 (40.0, 58.0)	.82	0.048
Lawsuit duration days, median (IQR)	222.3 (120.0, 423.0)	174.0 (100.0, 378.0)	.15	0.184
Lawsuit year, median (IQR)	2016.0 (2009.0, 2023.0)	2009.0 (2009.0, 2023.0)	.13	0.157
Accident severity grade, n (%)			.92	0.012
Grade 1/2	53 (14.7)	13 (14.3)		
Grade 3/4	307 (85.3)	78 (85.7)		
Court level, n (%)			.44	0.091
yes	146 (40.6)	41 (45.1)		
no	214 (59.4)	50 (54.9)		
Sex, n (%)			.48	.08
male	189 (52.5)	44 (48.4)		
female	171 (47.5)	47 (51.6)		
Disability severity grade, n (%)			.30	0.121
Grade 1-4	180 (50.0)	40 (44.0)		
Grade 5-10	180 (50.0)	51 (56.0)		
Tertiary hospital, n (%)			.83	0.025
yes	138 (38.3)	36 (39.6)		
no	222 (61.7)	55 (60.4)		

Inappropriate surgical procedure, n (%)			>.99	0.001
yes	190 (52.8)	48 (52.7)		
no	170 (47.2)	43 (47.3)		
Improper postoperative care, n (%)			.43	0.09
yes	181 (50.3)	50 (54.9)		
no	179 (49.7)	41 (45.1)		
Inadequate medical records, n (%)			.70	0.045
yes	170 (47.2)	45 (49.5)		
no	190 (52.8)	46 (50.5)		
Lack of informed consent, n (%)			.86	0.021
yes	194 (53.9)	50 (54.9)		
no	166 (46.1)	41 (45.1)		
Suboptimal surgical outcome, n (%)			.92	0.012
yes	299 (83.1)	76 (83.5)		
no	61 (16.9)	15 (16.5)		
Treatment delay, n (%)			.96	0.006
yes	187 (51.9)	47 (51.6)		
no	173 (48.1)	44 (48.4)		
Diagnostic error, n (%)			.97	0.004
yes	48 (13.3)	12 (13.2)		
no	312 (86.7)	79 (86.8)		
Improper complication management, n (%)			.18	0.157

yes	146 (40.6)	44 (48.4)		
no	214 (59.4)	47 (51.6)		
Inadequate preoperative preparation, n (%)			.50	0.078
yes	53 (14.7)	16 (17.6)		
no	307 (85.3)	75 (82.4)		
Unauthorized medical practice, n (%)			.45	0.09
yes	59 (16.4)	12 (13.2)		
no	301 (83.6)	79 (86.8)		
Improper medication use, n (%)			.22	0.14
yes	49 (13.6)	17 (18.7)		
no	311 (86.4)	74 (81.3)		
Breach of clinical standards, n (%)			.57	0.066
yes	62 (17.2)	18 (19.8)		
no	298 (82.8)	73 (80.2)		
Adverse outcome-related preexisting conditions, n (%)			.97	0.004
yes	47 (13.1)	12 (13.2)		
no	313 (86.9)	79 (86.8)		
Natural disease progression, n (%)			.91	0.014
yes	38 (10.6)	10 (11.0)		
no	322 (89.4)	81 (89.0)		
Patient non-adherence, n (%)			.60	0.061
yes	44 (12.2)	13 (14.3)		

no	316 (87.8)	78 (85.7)	

Values are presented as median (IQR) for continuous variables and n (%) for categorical variables. SMD: standardized mean difference.

Supplementary Table S13. MCC results across datasets.

Algorithm	Training MCC[a]	Internal test MCC	External validation MCC
LR[b]	0.578	0.753	0.731
KNN[c]	0.594	0.548	0.459
SVM[d]	0.817	0.578	0.524
RF[e]	0.621	0.684	0.650
XGBoost[f]	0.705	0.605	0.553

[a]MCC: Matthews correlation coefficient.

[b]LR: logistic regression.

[c]KNN: k-nearest neighbors.

[d]SVM: support vector machine.

[e]RF: random forest.

[f]XGBoost: extreme gradient boosting.

Supplementary Table S14. Grade Distribution.

Ordinal grade	Liability category	Compensation ratio interval, x (%)	Training, n (%)	Internal validation, n (%)	External validation, n (%)
0	No liability	x=0	11 (4.4)	5 (4.6)	5 (5.5)
1	Minor liability	0<x≤20	9 (3.6)	4 (3.7)	3 (3.3)
2	Secondary liability	20<x≤40	98 (39)	43 (39.4)	31 (34.1)
3	Equal liability	40<x≤60	44 (17.5)	19 (17.4)	22 (24.2)
4	Major liability	60<x≤90	68 (27.1)	29 (26.6)	26 (28.6)
5	Full liability	90<x≤100	21 (8.4)	9 (8.3)	4 (4.4)

Supplementary Table S15. Performance Metrics of Ordinal Liability-Grade Sensitivity Models.

Model	Dataset	MAE[a]	RMSE[b]	Exact accuracy[c]	Adjacent accuracy[d]	Quadratic weighted kappa[e]	Macro one-vs-rest AUROC[f]	AUROC for equal-or-higher liability grade[g]	Exploratory AUROC for original ≥50% endpoint[h]
Ordered logit[I]	Internal validation	0.752	1.173	0.505	0.789	0.455	0.710	0.895	0.906
Ordered logit	External validation	0.758	1.134	0.484	0.780	0.477	0.685	0.912	0.917
Ordered probit[J]	Internal validation	0.807	1.234	0.486	0.761	0.392	0.709	0.895	0.907
Ordered probit	External validation	0.758	1.134	0.484	0.780	0.476	0.690	0.915	0.918

[a]MAE: mean absolute error.

[b]RMSE: root mean squared error.

[c]Exact accuracy: proportion of exactly predicted grades.

[d]Adjacent accuracy: proportion of predictions within one grade of the observed grade.

[e]Quadratic weighted kappa: agreement measure for ordinal grades with greater penalties for larger disagreements.

[f]Macro one-vs-rest AUROC: average AUROC across liability grades.

[g]AUROC for equal-or-higher liability grade: discrimination at cumulative liability-grade thresholds.

[h]Exploratory AUROC for original ≥50% endpoint: discrimination for the original binary endpoint of compensation ratio ≥50%.

[I]Ordered logit: ordinal logistic regression model.

[J]Ordered probit: ordinal probit regression model.

Supplementary Table S16. Predictor Effects.

Model	Predictor	Beta[a]	Effect measure	Estimate[b]	95% CI[c]	*P value*	Direction
Ordered logit[d]	Age, per 10-year increase[e]	-0.429	Common OR[f]	0.65	0.55 to 0.77	<.001	Lower liability grade
Ordered logit	Inappropriate surgical procedure	1.397	Common OR	4.04	2.40 to 6.81	<.001	Higher liability grade
Ordered logit	Inadequate medical records	0.959	Common OR	2.61	1.56 to 4.37	<.001	Higher liability grade
Ordered logit	Lack of informed consent	0.744	Common OR	2.10	1.26 to 3.52	.005	Higher liability grade
Ordered logit	Disability severity grade 1-4	0.506	Common OR	1.66	0.99 to 2.78	.054	Higher liability grade
Ordered logit	Sex	0.693	Common OR	2.00	1.20 to 3.32	.008	Higher liability grade
Ordered logit	Treatment delay	0.406	Common OR	1.50	0.92 to 2.46	.11	Higher liability grade
Ordered logit	Inadequate preoperative preparation	-0.420	Common OR	0.66	0.33 to 1.32	.24	Lower liability grade
Ordered probit[g]	Age, per 10-year increase	-0.243	Coefficient	-0.243	-0.335 to -0.151	<.001	Lower liability grade
Ordered probit	Inappropriate surgical procedure	0.807	Coefficient	0.807	0.515 to 1.099	<.001	Higher liability grade
Ordered probit	Inadequate medical records	0.567	Coefficient	0.567	0.273 to 0.861	<.001	Higher liability grade
Ordered probit	Lack of informed consent	0.414	Coefficient	0.414	0.125 to 0.703	.005	Higher liability grade
Ordered probit	Disability severity grade 1-4	0.298	Coefficient	0.298	0.008 to 0.588	.04	Higher liability grade
Ordered probit	Sex	0.416	Coefficient	0.416	0.131 to 0.700	.004	Higher liability grade
Ordered probit	Treatment delay	0.191	Coefficient	0.191	-0.088 to 0.470	.18	Higher liability grade
Ordered probit	Inadequate preoperative preparation	-0.248	Coefficient	-0.248	-0.641 to 0.145	.22	Lower liability grade

[a]Beta: estimated regression coefficient.

[b]Estimate: effect-size estimate, presented as the common odds ratio for the ordered logit model and as the coefficient for the ordered probit model.

[c]CI: confidence interval.

[d]Ordered logit: ordinal logistic regression model.

[e]Age, per 10-year increase: age was scaled by 10 years, so the reported effect represents the change associated with each 10-year increase in age.

[f]OR: odds ratio.

[g]Ordered probit: ordinal probit regression model.

Supplementary Table S17. Included vs Excluded Cases.

Variable	Category	Included cases (n=451)	Excluded cases (n=58)	*P value*	SMD[a]
Age		50.5 (40.0, 58.0)	40.0 (40.0, 58.0)	.27	0.098
Lawsuit year		2013.0 (2009.0, 2023.0)	2017.0 (2009.0, 2023.0)	.49	-0.076
Lawsuit duration days		221.0 (120.0, 422.0)	163.0 (101.0, 395.0)	.39	0.074
Sex	Female	214 (48.3)	29 (50.9)	.82	-0.051
Sex	Male	229 (51.7)	28 (49.1)		0.051
Tertiary hospital	No	277 (61.4)	36 (62.1)	>.99	-0.013
Tertiary hospital	Yes	174 (38.6)	22 (37.9)		0.013
Court level	Other courts	264 (58.5)	41 (70.7)	.10	-0.256
Court level	Supreme/High People's Court	187 (41.5)	17 (29.3)		0.256
Region	Eastern China	195 (43.2)	15 (25.9)	.01	0.372
Region	Northeastern China	67 (14.9)	13 (22.4)		-0.195
Region	Central China	98 (21.7)	21 (36.2)		-0.323
Region	Western China	91 (20.2)	9 (15.5)		0.122
Accident severity grade	Grade 3/4	385 (85.4)	49 (84.5)	>.99	0.025
Accident severity grade	Grade 1/2	66 (14.6)	9 (15.5)		-0.025
Disability severity grade	Grade 5-10	231 (51.2)	28 (48.3)	.78	0.059
Disability severity grade	Grade 1-4	220 (48.8)	30 (51.7)		-0.059

[a]SMD: standardized mean difference.

Supplementary Table S18. IPW Diagnostics.

Panel A. Weight diagnostics.

Metric	Value
Marginal probability of inclusion	0.8861
Raw weight 1st percentile	0.9276
Raw weight 99th percentile	1.1730
Normalized stabilized IPW[a], mean	1.0000
Normalized stabilized IPW, SD	0.0601
Normalized stabilized IPW, min	0.9277
Normalized stabilized IPW, max	1.1731
Effective sample size among included cases	449.4

[a]IPW: inverse probability weighting.

Panel B. Covariate balance before and after IPW.

Variable	Category	SMD[a] before IPW[b]	SMD after IPW
Region	Eastern China	0.372	0.333
Region	Central China	-0.323	-0.284
Court level	Supreme/High People's Court	0.256	0.228
Region	Northeastern China	-0.195	-0.174
Region	Western China	0.122	0.109
Age	-	0.098	0.086
Lawsuit year	-	-0.076	-0.067
Lawsuit duration days	-	0.074	0.066
Disability severity grade	Grade 1-4	-0.059	-0.052
Sex	Male	0.051	0.046
Accident severity grade	Grade 1/2	-0.025	-0.021
Tertiary hospital	Yes	0.013	0.012

[a]SMD: standardized mean difference.

[b]IPW: inverse probability weighting.

Supplementary Table S19. IPW Sensitivity Results.

Dataset	Analysis	AUROC[c] (95% CI[d])	Brier score	Accuracy	Recall	Specificity	Precision
Internal test	Primary LR[a], unweighted evaluation	0.933 (0.883-0.972)	0.105	0.872	0.810	0.941	0.940
	Primary LR, IPW[b]-weighted evaluation	0.934 (0.884-0.972)	0.105	0.871	0.809	0.941	0.940
	IPW-refit LR, IPW-weighted evaluation	0.934 (0.885-0.973)	0.105	0.871	0.809	0.941	0.940
External validation	Primary LR, unweighted evaluation	0.915 (0.853-0.962)	0.119	0.857	0.776	0.952	0.950
	Primary LR, IPW-weighted evaluation	0.915 (0.854-0.962)	0.119	0.856	0.774	0.952	0.950
	IPW-refit LR, IPW-weighted evaluation	0.917 (0.857-0.963)	0.119	0.856	0.774	0.952	0.950

[a]LR: logistic regression.

[b]IPW: inverse probability weighting.

[c]AUROC: area under the receiver operating characteristic curve.

[d]CI: confidence interval.

Supplementary Table S20. Temporal Subgroup Performance.

Period	N	High compensation ratio ≥50%, n (%)	AUROC[a] (95% CI[b])	Brier score	Accuracy	Recall	Specificity	Precision	F1 score
2004-2020	197	99 (50.3)	0.903 (0.856-0.939)	0.131	0.817	0.859	0.776	0.794	0.825
2021-2025	163	87 (53.4)	0.902 (0.853-0.944)	0.125	0.834	0.874	0.789	0.826	0.849

The AUROC difference between the 2021-2025 and 2004-2020 periods was minimal: ΔAUROC=-0.001.

[a]AUROC: area under the receiver operating characteristic curve.

[b]CI: confidence interval.

Supplementary Table S21. Logistic regression models with temporal adjustment.

Variable	Refitted core model OR[a] (95% CI[b]), *P value*	Post-2021 adjusted OR (95% CI), *P value*	Lawsuit year-adjusted OR (95% CI), *P value*
Sex	2.742 (1.489-5.048), *P*=.001	2.744 (1.490-5.053), *P*=.001	2.748 (1.491-5.063), *P*=.001
Age, per 10-year increase[c]	0.490 (0.382-0.628), *P*<.001	0.490 (0.382-0.629), *P*<.001	0.490 (0.382-0.628), *P*<.001
Disability severity grade 1-4	3.060 (1.624-5.768), *P*<.001	3.094 (1.636-5.853), *P*<.001	3.076 (1.626-5.820), *P*<.001
Inappropriate surgical procedure	8.227 (4.340-15.596), *P*<.001	8.292 (4.366-15.746), *P*<.001	8.253 (4.348-15.666), *P*<.001
Inadequate medical records	3.648 (1.960-6.788), *P*<.001	3.657 (1.964-6.811), *P*<.001	3.646 (1.959-6.786), *P*<.001
Lack of informed consent	3.747 (2.036-6.898), *P*<.001	3.709 (2.009-6.846), *P*<.001	3.729 (2.019-6.887), *P*<.001
Treatment delay	2.487 (1.348-4.591), *P*=.004	2.490 (1.349-4.599), *P*=.004	2.489 (1.348-4.596), *P*=.004
Inadequate preoperative preparation	0.482 (0.205-1.137), *P*=.10	0.487 (0.206-1.149), *P*=.10	0.484 (0.205-1.144), *P*=.10
Post-2021 legal period	-	0.900 (0.487-1.664), *P*=.74	-
Lawsuit year, per 1-year increase	-	-	0.997(0.952-1.043), *P*=.88

[a]OR: odds ratio;

[b]CI: confidence interval.

[c]Age, per 10-year increase: age was scaled by 10 years, so the reported effect represents the change associated with each 10-year increase in age.

The post-2021 legal-period indicator was coded according to whether the lawsuit year was 2021 or later. The lawsuit year was entered as a continuous centered variable in the lawsuit year-adjusted model.

Supplementary Table S21 presents post hoc temporal sensitivity analyses based on logistic regression models refitted using the full development cohort (n=360). These models used the same core predictors as the primary model presented in Figure 4 but were fitted in a different analytical cohort. Temporal effects were examined using alternative specifications of lawsuit year, including a post-2021 legal-period indicator and a continuous centered lawsuit-year variable. The refitted core model OR column represents the baseline model within the temporal sensitivity analysis framework and should not be interpreted as the primary model estimates shown in Figure 4.
