# Supplementary material for: Development and Validation of Machine Learning Models for Postjudgment Estimation of High Compensation Ratios After Lower Limb Fracture Surgery: Retrospective Study
Source: JMIR Med Inform. 2026 Sep 22;14:e93801. doi: 10.2196/93801 (PMC13643259; doi:10.2196/93801)
Supplement: Supplementary file 3 [file medinform_v14i1e93801_app3.pdf]

Supplementary Figures

Figure S1 Selection of the optimal penalization parameter (α)................................2

Figure S2 LASSO coefficient profiles...3

Figure S3 Coefficients of the features selected by LASSO.......................................4

Figure S4 ROC curves of the models in the expanded hyperparameter sensitivity analysis...5

Figure S5 Comprehensive evaluation of model performance on the external validation set...6

Figure S6 Distribution of inclusion probabilities and IPW weights........................7

Figure S7 Temporal subgroup ROC curves..8

Figure S8 Temporal subgroup calibration curves..9

Figure S9 Interface of the web-based post-judgment medicolegal reference assessment tool...10

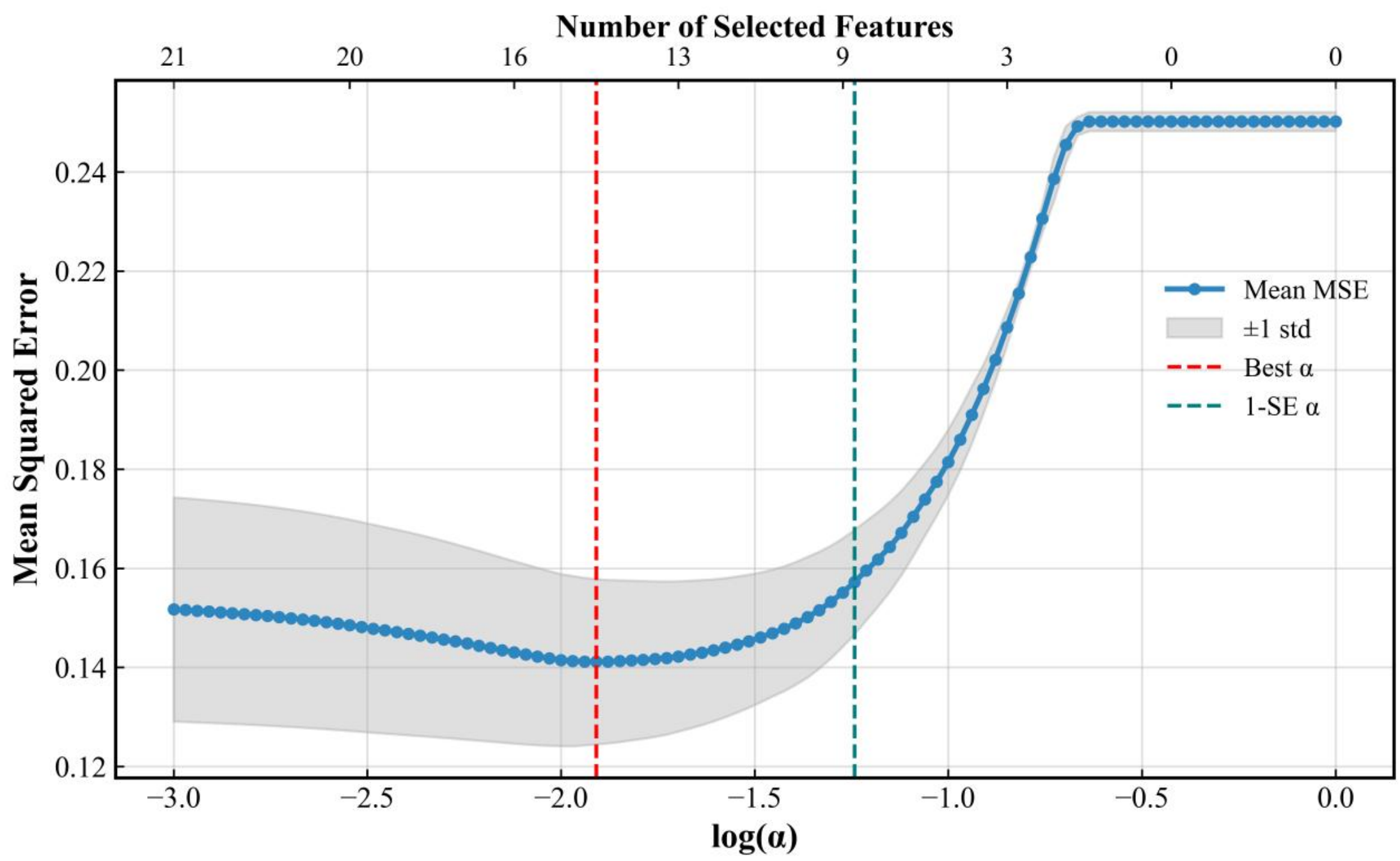

Supplementary Figure S1. Selection of the optimal penalization parameter (α). The plot displays the Mean Squared Error (MSE) versus log(α) using 5-fold cross-validation. The vertical dotted lines represent the minimum MSE (α_{min}) and the 1-standard-error criterion (α_{1SE}).

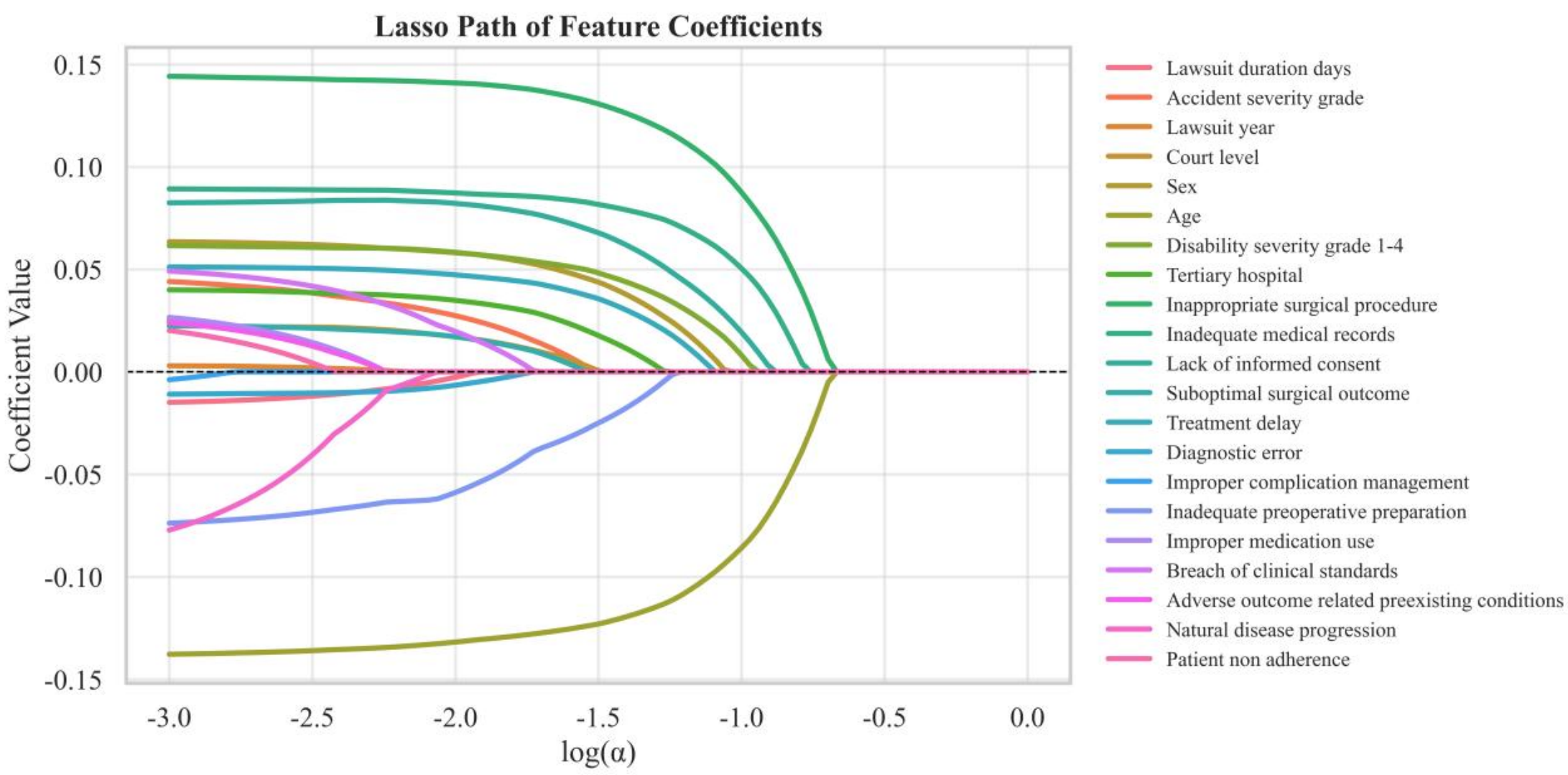

Supplementary Figure S2. LASSO coefficient profiles.

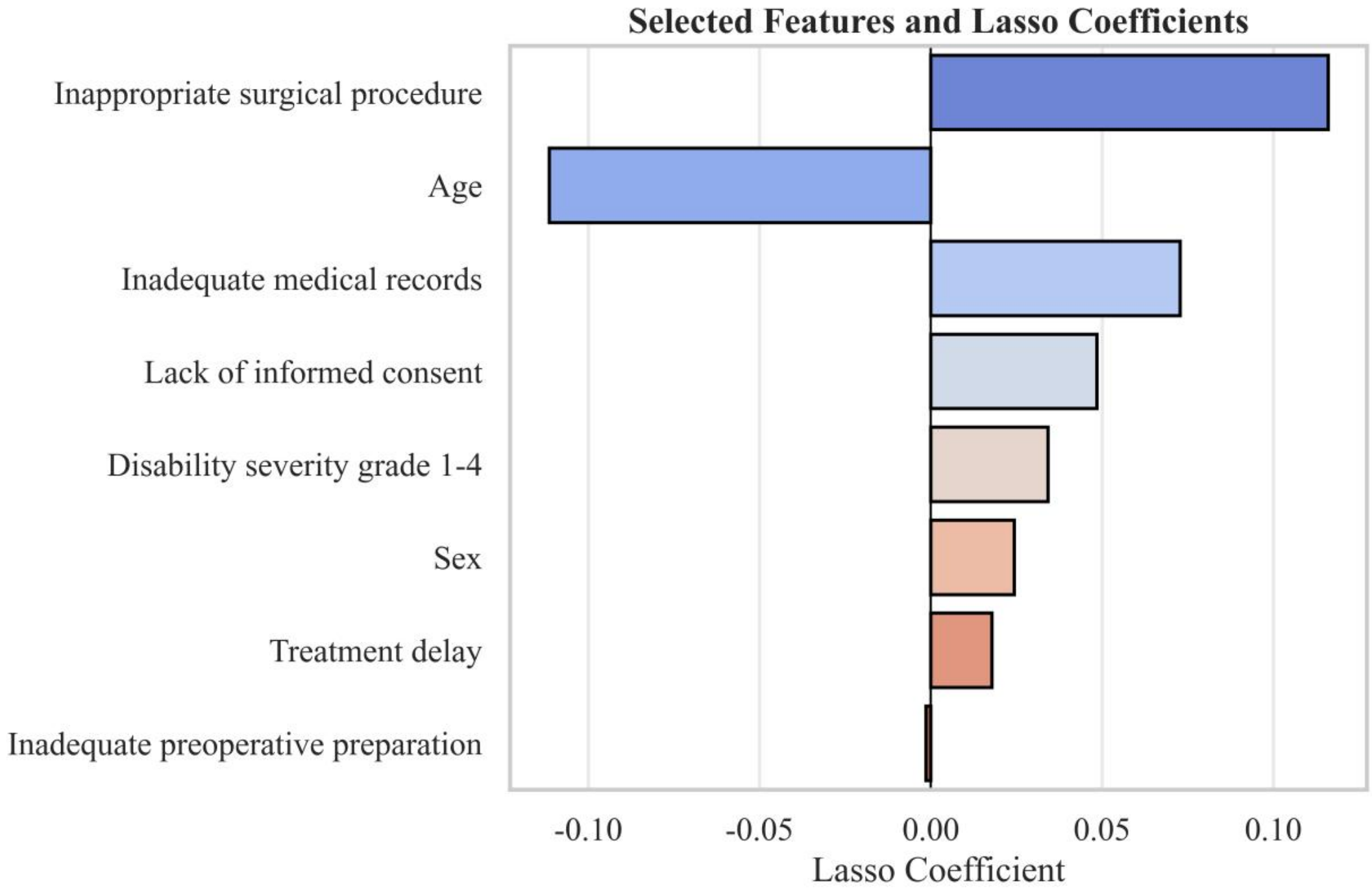

Supplementary Figure S3. Coefficients of the features selected by LASSO.

A

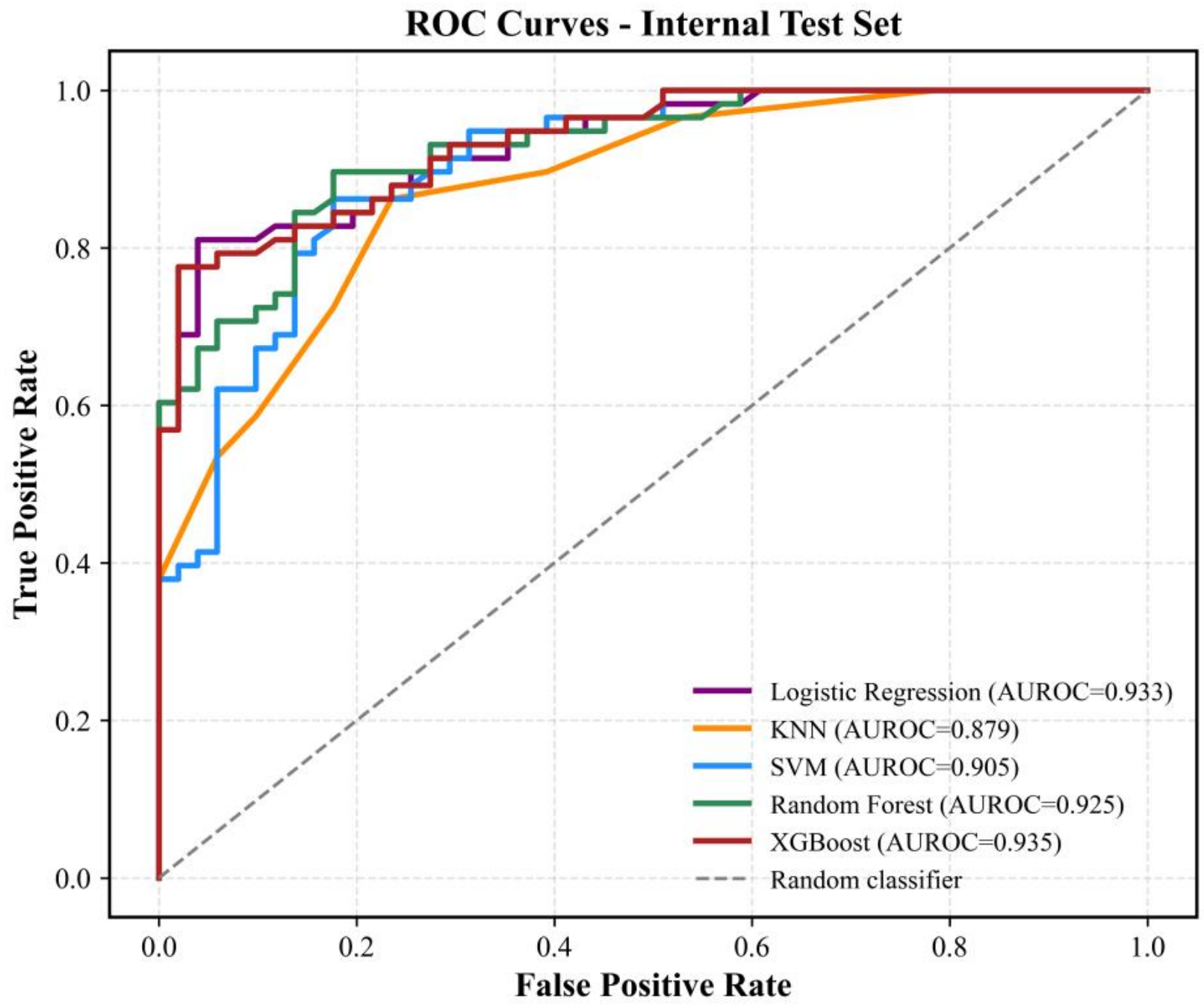

B

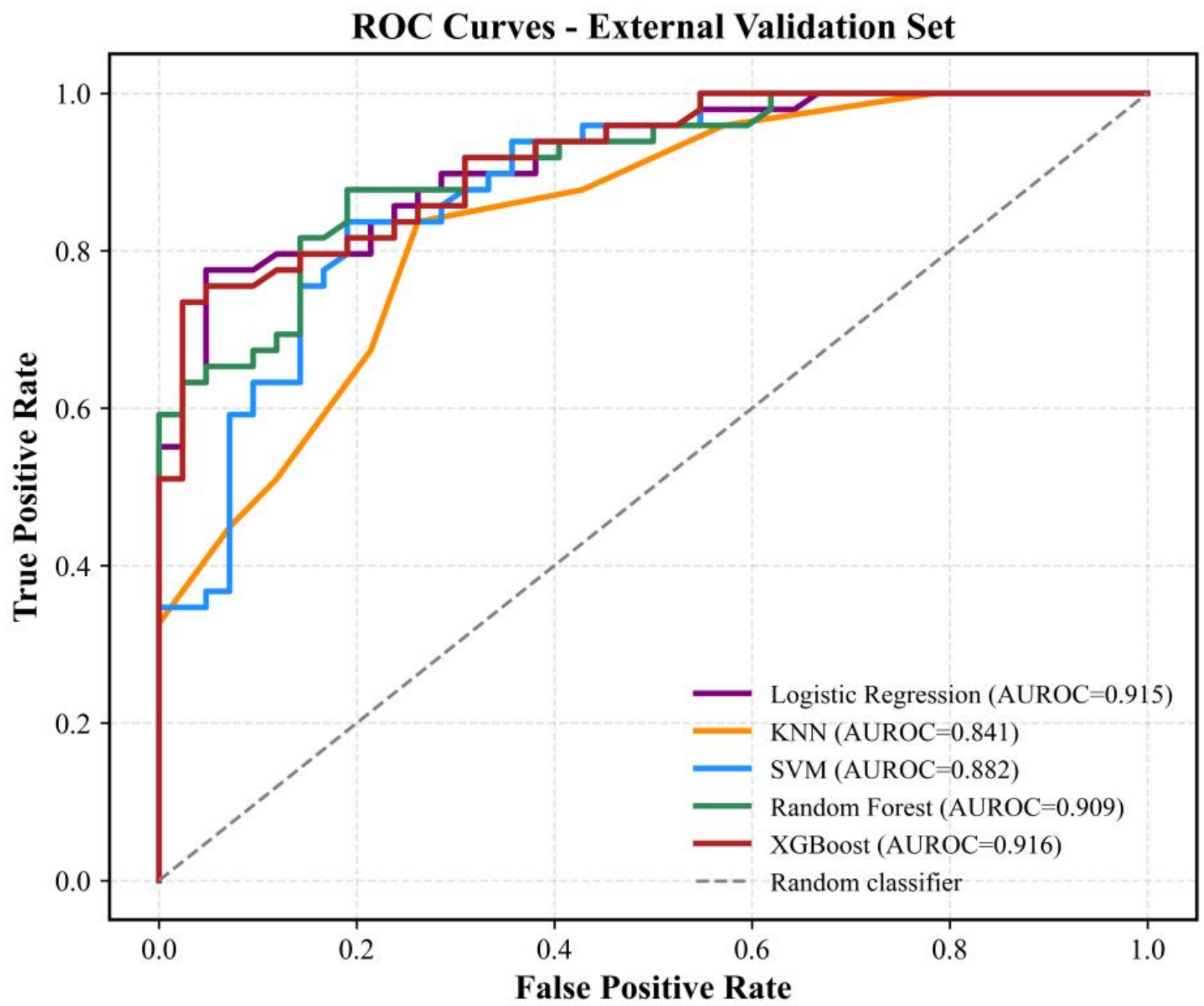

Supplementary Figure S4. ROC curves of the models in the expanded hyperparameter sensitivity analysis. (A) Internal test set. (B) External validation set. The gray dashed line represents the random classifier.

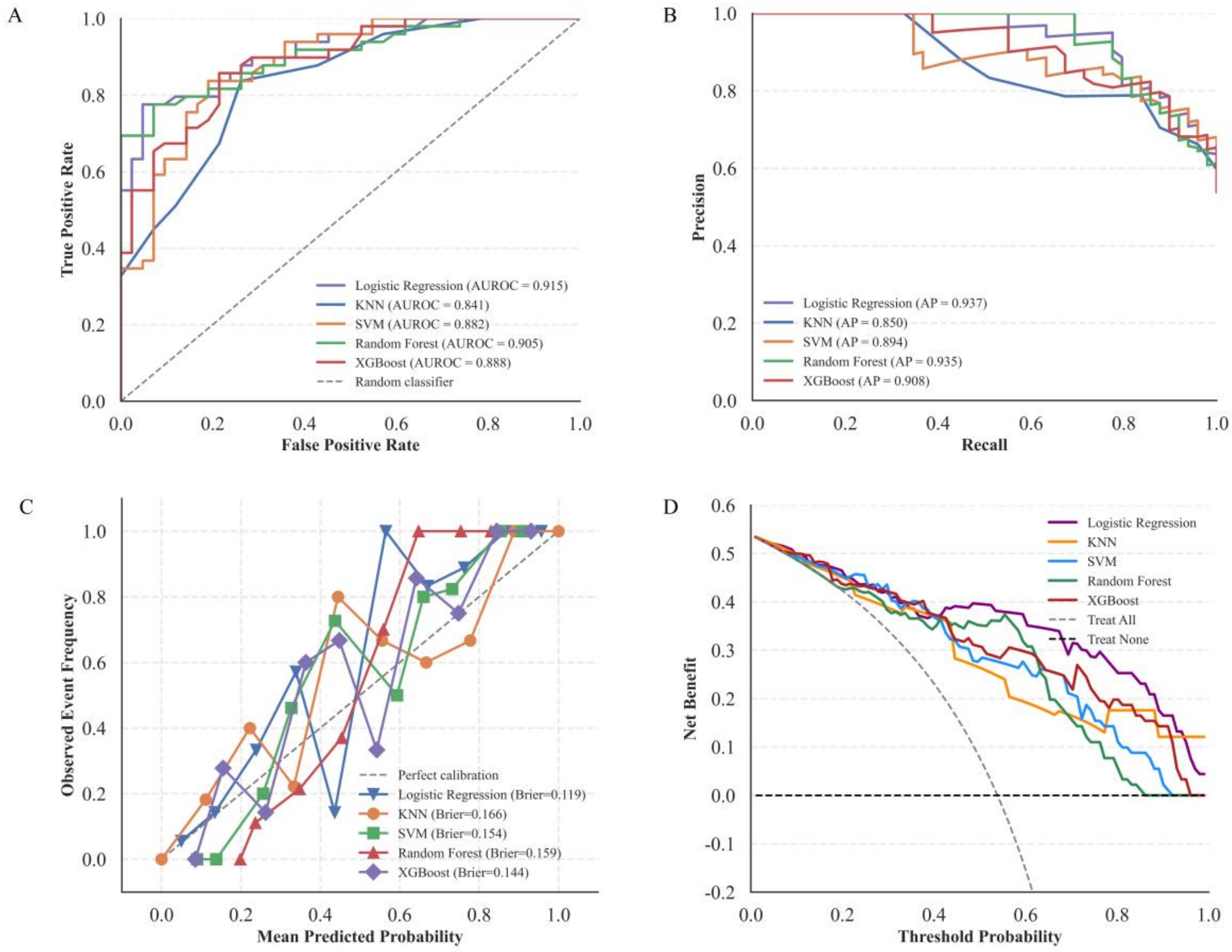

Supplementary Figure S5. Comprehensive evaluation of model performance on the external validation set.

A

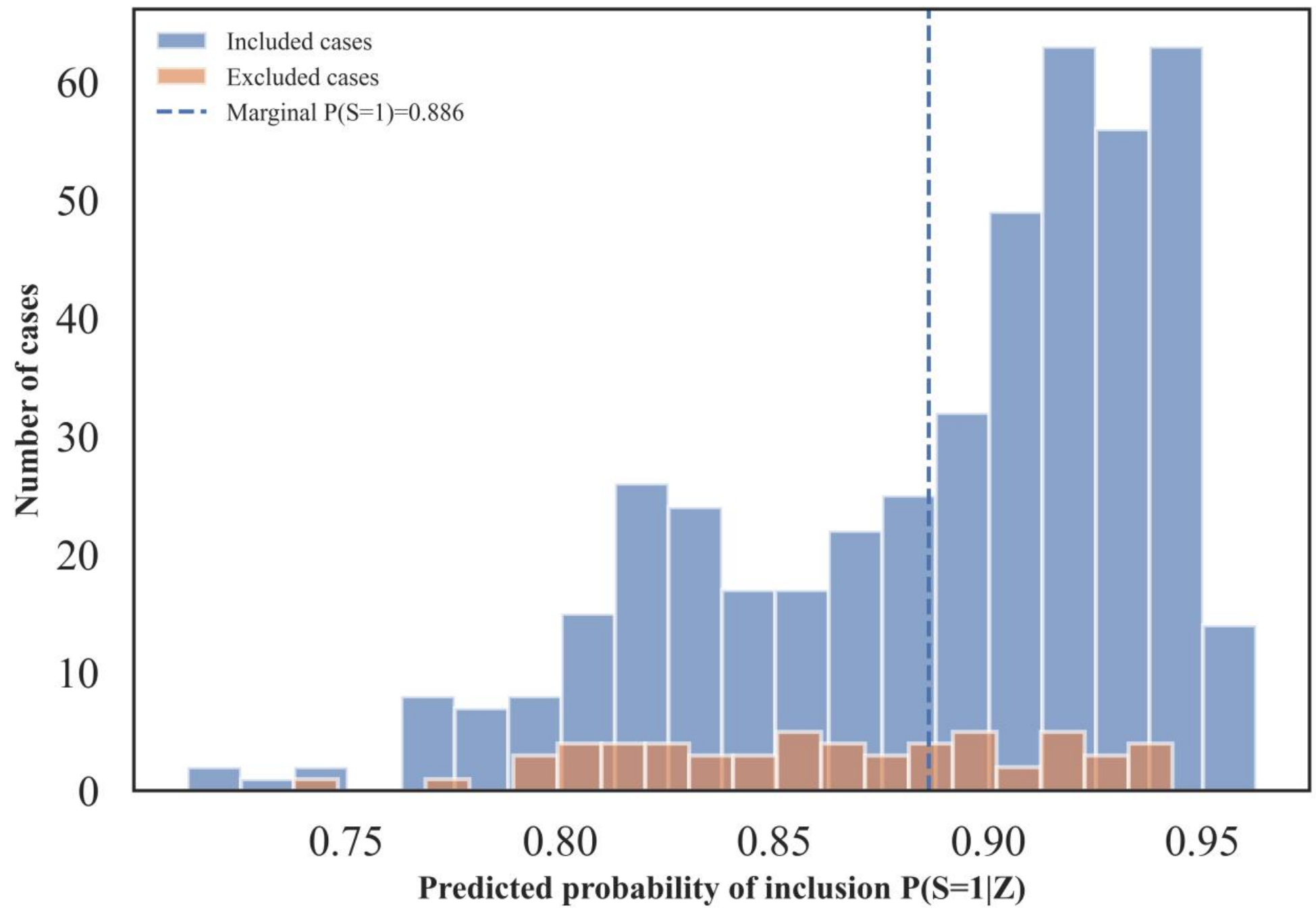

B

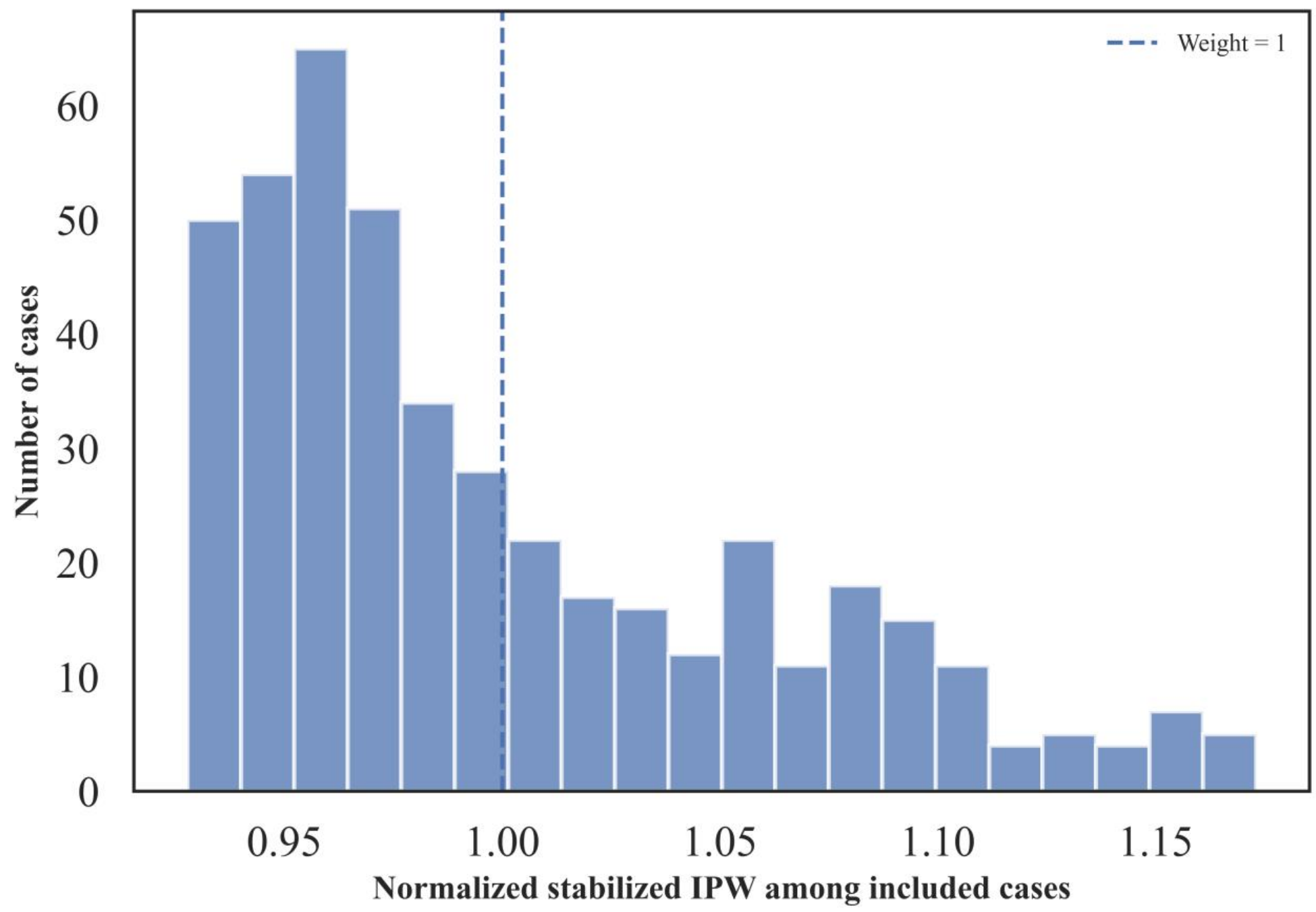

Supplementary Figure S6. Distribution of inclusion probabilities and IPW weights. (A) Predicted probability of inclusion among included and excluded cases. The dashed line indicates the marginal probability of inclusion. (B) Distribution of normalized stabilized inverse probability weights among included cases. The dashed line indicates weight=1. IPW: inverse probability weighting.

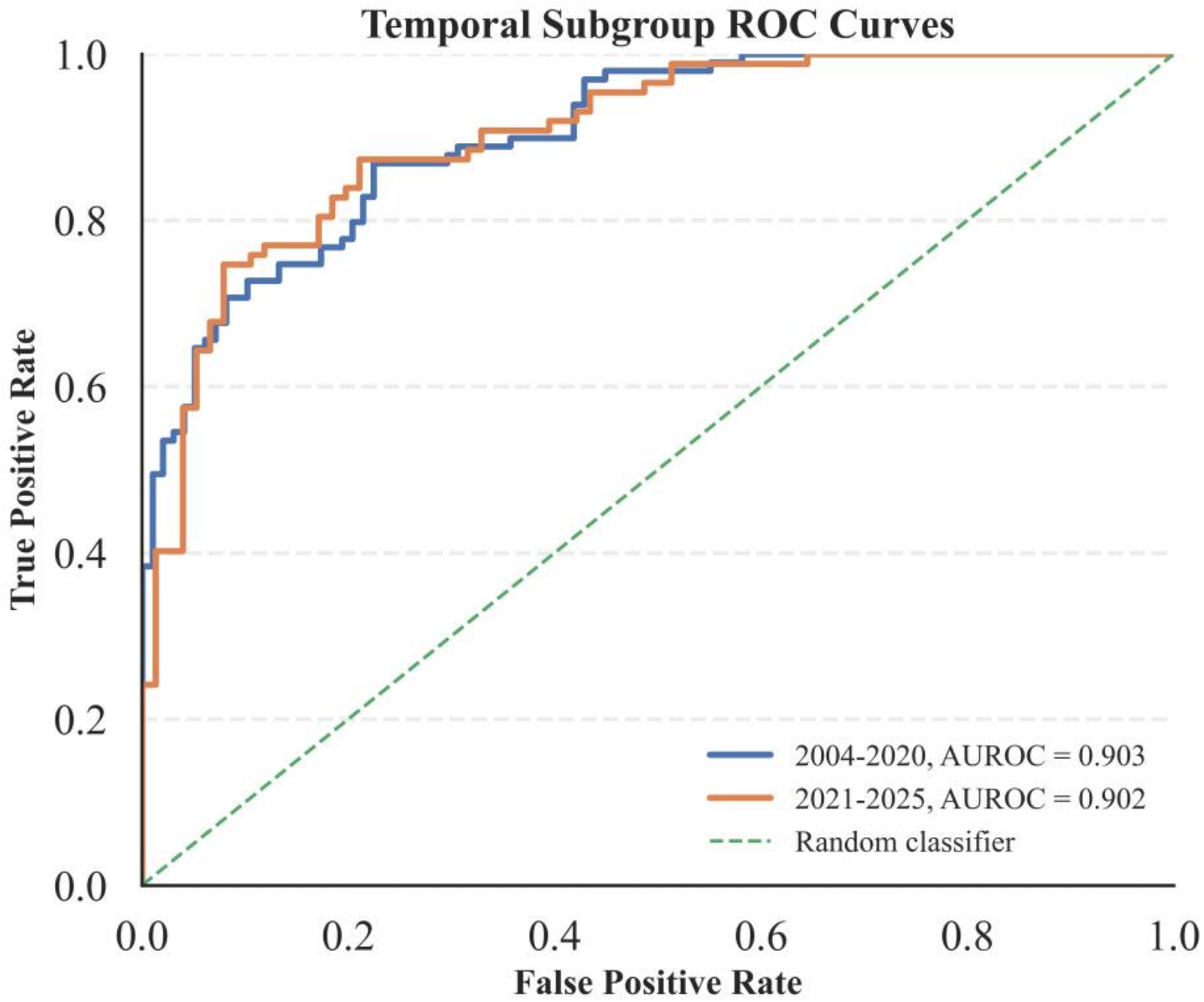

Supplementary Figure S7. Temporal subgroup ROC curves. Receiver operating characteristic curves of the final logistic regression model in the 2004-2020 and 2021-2025 temporal subgroups. The dashed diagonal line represents a random classifier.

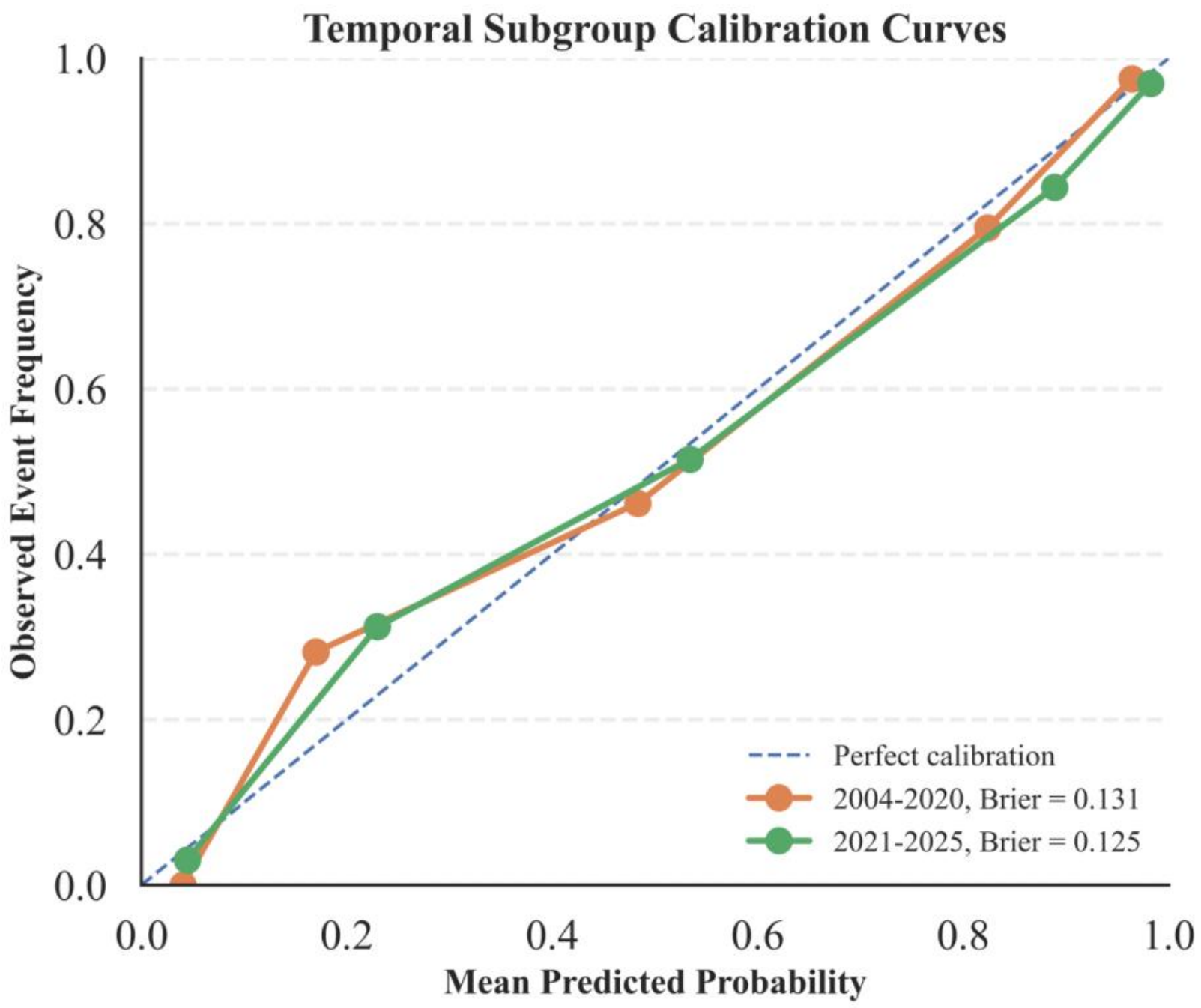

Supplementary Figure S8. Temporal subgroup calibration curves. Calibration curves of the final logistic regression model in the 2004-2020 and 2021-2025 temporal subgroups. The dashed diagonal line represents perfect calibration. Brier scores were 0.131 for 2004-2020 and 0.125 for 2021-2025.

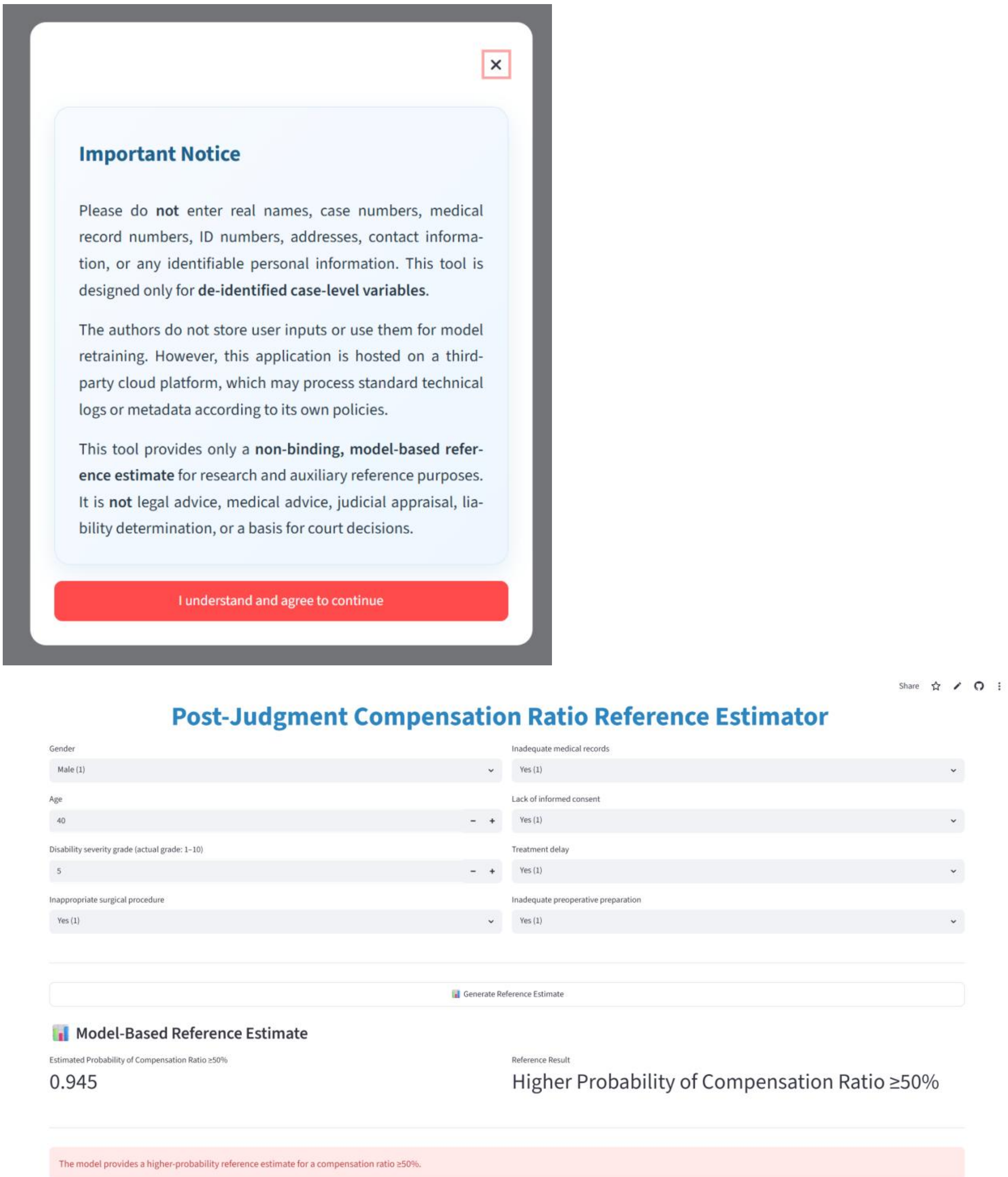

Supplementary Figure S9. Interface of the web-based post-judgment medicolegal reference assessment tool. Available at https://compensation-ratio-reference-estimator.streamlit.app/.
